# Polyfunctional Sterically Hindered Catechols with Additional Phenolic Group and Their Triphenylantimony(V) Catecholates: Synthesis, Structure, and Redox Properties

**DOI:** 10.3390/molecules25081770

**Published:** 2020-04-12

**Authors:** Ivan V. Smolyaninov, Andrey I. Poddel’sky, Susanna A. Smolyaninova, Maxim V. Arsenyev, Georgy K. Fukin, Nadezhda T. Berberova

**Affiliations:** 1 Toxicology Research Group, Federal State Budgetary Institution of Science “Federal Research Centre The Southern Scientific Centre of the Russian Academy of The Sciences”, Tatischeva str. 16, 414056 Astrakhan, Russia; ivsmolyaninov@gmail.com; 2 Department of Chemistry, Astrakhan State Technical University, 16 Tatisheva str., Astrakhan 414056, Russia; zeynalovasa@mail.ru (S.A.S.); nberberova@gmail.com (N.T.B.); 3 G.A. Razuvaev Institute of Organometallic Chemistry, Russian Academy of Sciences, 49 Tropinina str., 603137 Nizhny Novgorod, Russia; mars@iomc.ras.ru (M.V.A.); gera@iomc.ras.ru (G.K.F.)

**Keywords:** redox-active ligand, catechol, thioether, antimony, X-ray, cyclic voltammetry, electronic paramagnetic resonance

## Abstract

New polyfunctional sterically hindered 3,5-di-*tert*-butylcatechols with an additional phenolic group in the sixth position connected by a bridging sulfur atom—(6-(CH_2_-S-tBu_2_Phenol)-3,5-DBCat)H_2_ (**L_1_**), (6-(S-tBu_2_Phenol)-3,5-DBCat)H_2_ (**L_2_**), and (6-(S-Phenol)-3,5-DBCat)H_2_ (**L_3_**) (3,5-DBCat is dianion 3,5-di-*tert*-butylcatecolate)—were synthesized and characterized in detail. The exchange reaction between catechols **L_1_** and **L_3_** with triphenylantimony(V) dibromide in the presence of triethylamine leads to the corresponding triphenylantimony(V) catecholates (6-(CH_2_-S-tBu_2_Phenol)-3,5-DBCat)SbPh_3_ (**1**) and (6-(S-Phenol)-3,5-DBCat)SbPh_3_ (**2**). The electrochemical properties of catechols **L_1_**–**L_3_** and catecholates **1** and **2** were investigated using cyclic voltammetry. The electrochemical oxidation of **L_1_**–**L_3_** at the first stage proceeds with the formation of the corresponding *o*-benzoquinones. The second process is the oxidation of the phenolic moiety. Complexes **1** and **2** significantly expand their redox capabilities, owing to the fact that they can act as the electron donors due to the catecholate metallocycle capable of sequential oxidations, and as donors of the hydrogen atoms, thus forming a stable phenoxyl radical. The molecular structures of the free ligand **L_1_** and complex **1** in the crystal state were determined by single-crystal X-ray analysis.

## 1. Introduction

A large amount of data has been accumulated to the present time on the complexes of transition and non-transition elements with redox active ligands: *o*-benzoquinones, *o*-iminobenzoquinones and alpha-diimines [1,2,3,4,5,6,7,8,9,10,11]. A feature of these compounds is the participation of not only metal but also redox-active ligands in the processes of electron transfer and, as a result, they are capable of reversible accepting and donating electrons during the different chemical transformations, while remaining bound to the metal. This feature can significantly expand or change the reactivity of metal complexes. Studies of non-transition metal complexes containing redox-active ligands of the *o*-quinonato type have allowed to reveal an expansion of the valence potentials of non-transition metals due to redox-active ligands [12,13], to observe of the phenomenon of redox isomerism in the main group metals’ chemistry [14], and also to observe and investigate the ability of antimony(V) complexes to reversible fixation of molecular oxygen [15,16,17,18,19], to observe the fixation of nitrogen(II) oxide (by zinc(II) and lead(II) catecholates) [20].

There are several approaches that allow to change the properties of ligands and their metal complexes: (1) a modification of the ligands’ structure by the variation of heteroatoms (O, N, S) or the presence of bulky organic substituents; (2) the introduction of different electron donor/acceptor groups or additional redox-active as well as chelating fragments at various positions of the quinoid ring; and (3) a combination of a redox-active ligand and an additional coordination center. Organic and organometallic compounds containing several redox centers are of particular interest in view of a number of their specific features: the possibility of indirect activation through certain functional groups, intramolecular electron transfer, proton-conjugated electron transfer, etc. From the example of ferrocene derivatives (ferrocifenes) as well as quinoid compounds, it was shown that various types of biological activities (cytotoxicity, antibacterial, antiparasitic, antioxidant) depend directly on the presence of a redox-active group and its transformations [21,22,23,24]. Redox-asymmetric systems can be built not only on the basis of a ferrocenyl fragment but also on a combination of phenolic, amide, and amino groups, which are also of interest because of the possibility of proton-conjugated charge transfer reactions [25,26,27].

For a long time, dioxolenes have attracted the attention of researchers because of the possibility of their existence in several redox states. Recently, the focus of research in this area has been aimed at their functionalization by introducing additional redox-active groups [28,29,30,31] or coordination sites [32,33,34,35]. In the series of functionalized derivatives of catechol/*o*-quinones, sulfur-containing compounds occupy a special place since sulfide bridge fragments also exhibit redox activity. The introduction of the tetrathiafulvalene linker allows to obtain bis-quinones and their metal complexes which exhibit unusual physicochemical properties [36,37,38,39]. The sterically hindered dithiete-annelated *o*-benzoquinone is a bifunctional ligand, which combines two chalcogen redox-active coordination centers in its structure—dioxolene and dithiolene—allowing it to form complexes in which various coordination sites are involved [40]. S-functionalization of 3,5-di-*tert*-butyl-*o*-benzoquinone with cystamine and cysteine has made it possible to obtain Cu(II), Zn(II), and Ni(II) complexes [41,42]. A similar reaction with dithiols makes it possible to synthesize catechols with a free thio group or bis-catechol thioethers [43,44], which have chelating properties and the ability to be absorbed on the surface [45,46,47]. Variation of the organic groups in thiols allows one to obtain a wide range of thiolated catechols, which exhibit antioxidant, antiradical, and cryoprotective activity [48,49,50]. Transition metal compounds based on sulfur-containing catecholate/*o*-semiquinolate ligands demonstrate antifungal and antibacterial activity [51,52]. Thus, the synthesis and study of the properties of thiolated dioxiolene ligands and metal complexes based on them is relevant in view of the diversity of the properties manifested. Antimony(III/V) derivatives are used as antiparasitic agents in the treatment of leishmaniasis [53], and have potential as antitumor, cytotoxic, and antibacterial substances [54,55]. Along with biological activity, antimony complexes are promising compounds for the design of functional materials. The presence of a catecholate cycle at the antimony atom allows the creation of new colorimetric and fluorescent sensors for the fluoride anion [56,57,58,59]. The polymer compositions containing catecholate derivatives of triphenylantimony(V) are promising for the creation of polymeric materials capable of fixing oxygen [60,61,62].

In the present paper, we report on new thioethers combining several redox-active centers: A chelating catechol fragment, a phenolic group, and a thioether linker. The coordinating activity of the catechol fragment was studied in a reaction with triphenylantimony(V) dibromide, and as a result, triphenylantimony(V) catecholates, which contain redox-active centers of a different nature, were synthesized. The molecular structure and electrochemical transformations of the resulting compounds are discussed.

## 2. Results and Discussion

### 2.1. Synthesis and Characterization

**Scheme 1:**
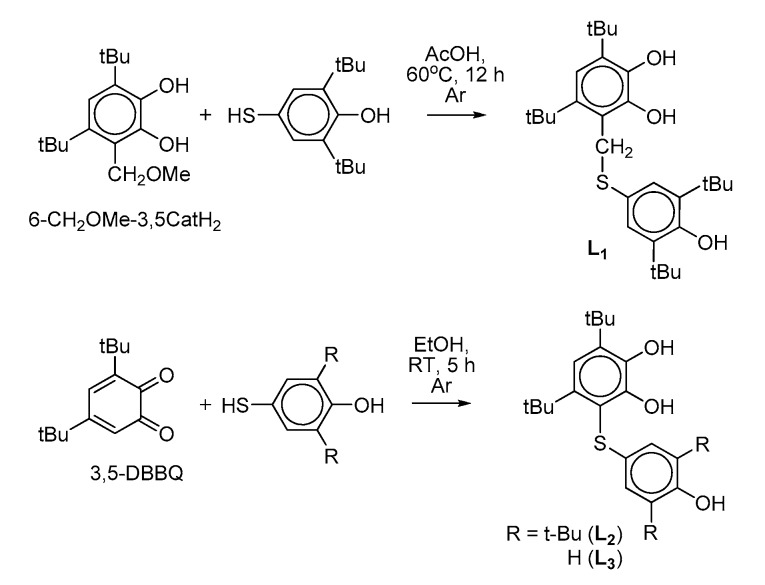
Synthesis of catechols **L_1_**–**L_3_**.

The sterically hindered catechol (6-(CH_2_-S-tBu_2_Phenol)-3,5-DBCat)H_2_ (**L_1_**) was synthesized from 6-methoxymethyl-3,5-di-*tert*-butylcatechol (6-CH_2_OMe-3,5-Cat)H_2_ and 2,6-di-*tert*-butyl-4-mercaptophenol in a solution of acetic acid at 60°C (Scheme 1(1)). The 3,5-di-*tert*-butylcatechols (6-(S-tBu_2_Phenol)-3,5-DBCat)H_2_ (**L_2_**) and (6-(S-Phenol)-3,5-DBCat)H_2_ (**L_3_**) were prepared by the reaction of 3,5-di-*tert*-butyl-*o*-benzoquinone with 2,6-di-*tert*-butyl-4-mercaptophenol or 4-mercaptophenol [48], respectively, in ethanol solution (Scheme 1(2)).

Catechols **L_1_**, **L_2_** were isolated as pale-yellow microcrystalline powders that were well-soluble in toluene or chloroform. The composition of these catechols was determined by means of IR-, ^1^H, and ^13^C{^1^H} NMR spectroscopy and elemental analysis. A prolonged recrystallization of **L_1_** from n-hexane allowed X-ray-quality white crystals of **L_1_** to be grown.

**Scheme 2:**
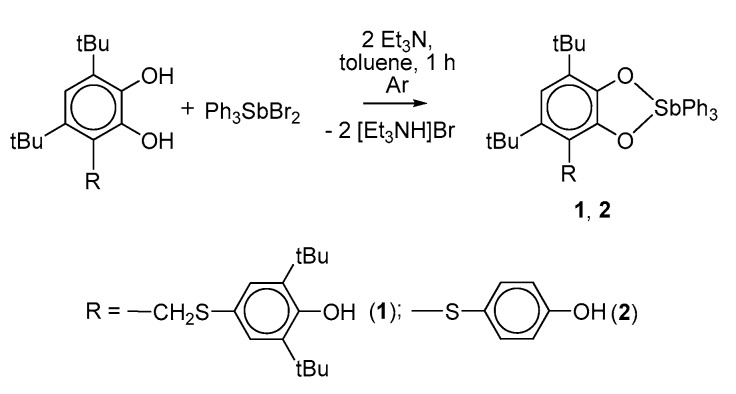
Synthesis of complexes **1** and **2**.

A substitution reaction between catechols **L_1_** or **L_3_** and triphenylantimony(V) dibromide in the presence of two equivalents of triethylamine in toluene solution leads to the corresponding triphenylantimony(V) catecholates (Scheme 2).

Complexes **1** and **2** were isolated as yellow solids. These compounds are readily soluble in common organic solvents, such as aliphatic or aromatic hydrocarbons, chloroform, ethers, and methanol. Complexes **1** and **2** were characterized by IR-, ^1^H, and ^13^C{^1^H} NMR spectroscopy and elemental analysis. The X-ray suitable crystals of **1** were grown by the prolonged (during 5 days) crystallization of complex in toluene solution at −18°C.

### 2.2. Molecular Structures

**Figure 1:**
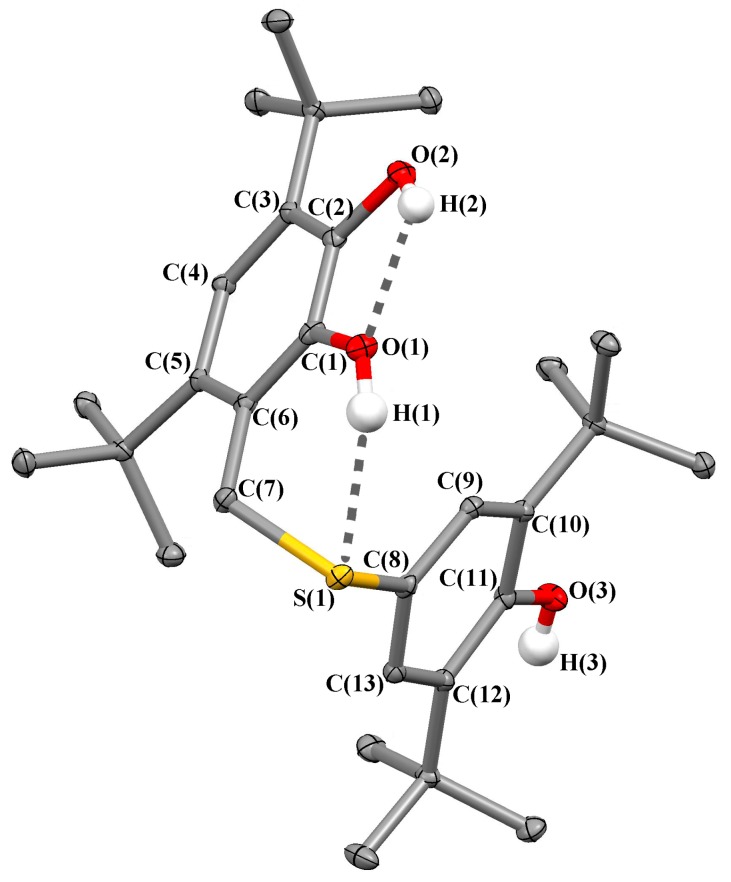
The X-ray structure of catechol **L_1_** (6-(CH_2_-S-tBu_2_Phenol)-3,5-DBCat)H_2_ in crystal. The hydrogen atoms except hydroxyl hydrogens are omitted for clarity. The ellipsoids of 50% probability. The selected bond distances (Å): O(1)-C(1) 1.3816(14), O(2)-C(2) 1.3804(14), O(3)-C(11) 1.3806(14), S(1)-C(7) 1.8527(12), S(1)-C(8) 1.7765(11), C(1)-C(2) 1.3967(16), C(1)-C(6) 1.3987(16), C(2)-C(3) 1.3910(16), C(3)-C(4) 1.4029(15), C(4)-C(5) 1.4016(15), C(5)-C(6) 1.4150(15), C(6)-C(7) 1.5101(15), C(8)-C(9) 1.3907(16), C(8)-C(13) 1.3893(16), C(9)-C(10) 1.3947(16), C(10)-C(11) 1.4118(15), C(11)-C(12) 1.4139(15), C(12)-C(13) 1.3949(16).

The molecular structure of catechol **L_1_** in crystal is shown on Figure 1. The general geometrical parameters of catechol fragment are typical for this class of compounds. The carbon–carbon bonds in a ring C(1-6) have an average distance of 1.401 ± 0.014 Å, which is typical of aromatic systems. The carbon–oxygen distances are ordinary [63] and close to each other: the bonds O(1)-C(1), O(2)-C(2), and O(3)-C(11) are 1.3816(14), 1.3804(14), and 1.3806(14) Å, respectively. The molecule is not planar, the angle between catechol and phenolic aromatic rings planes is equal to 54.7(1)°, the torsion angle is 47.9(1)°, and the torsion angle C(1)C(6)C(7)S(1) is 56.7(1)°. Such distortion of geometry is supported by an intramolecular hydrogen bonding in the molecule: the distance O(2)-H(2)…O(1) is 2.07(1) Å, angle O(2)-H(2)-O(1) is 126.6(1)°, and the distance O(1)-H(1)…S(1) is 2.28(1) Å, angle O(1)-H(1)-S(1) is 153.0(1)°. In the crystal, molecules form pairs due to the intermolecular hydrogen bonding between phenolic hydroxyl groups (Figure S9). The intermolecular distances O(3)-H(3)…O(3′) are 1.71(1) Å, and the corresponding angles O(3)-H(3)-O(3′) are 113.5(1)°. The distance between the planes of catecholate rings in neighboring pairs is 3.49(1) Å, which is close to the sum of the Van der Waals radii of carbon atoms [64].

**Figure 2:**
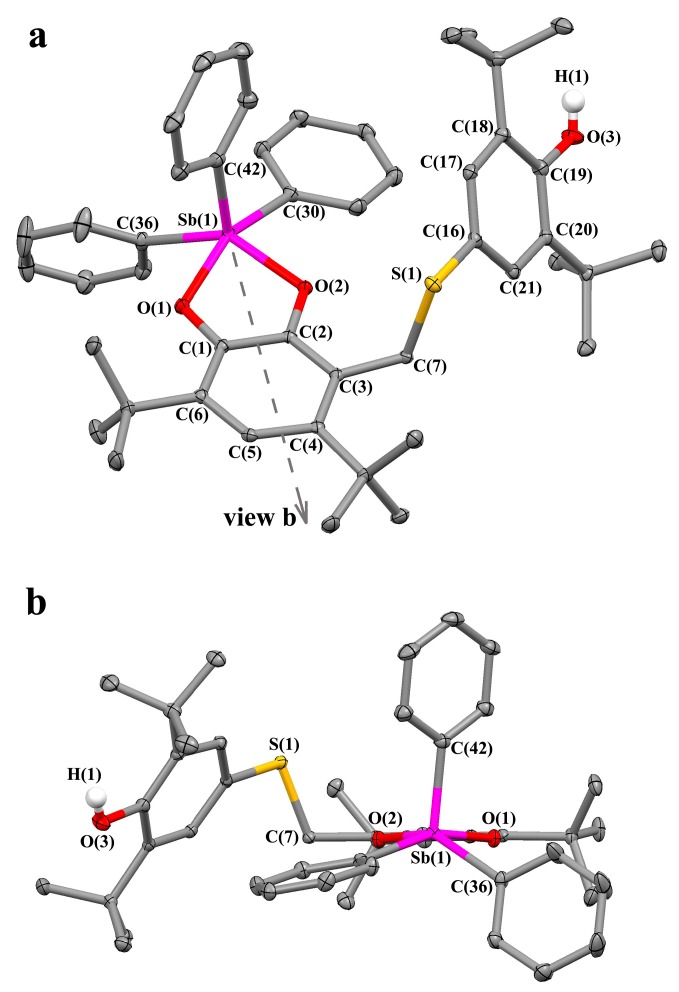
The X-ray structure of **1**: (**a**) the general view on molecule of **1**; (**b**) the view on molecule **1** along the dotted line (view b) depicted on Figure 2a. The hydrogen atoms except hydroxyl hydrogen H(1) are omitted for clarity. The ellipsoids of 50% probability. The selected bond distances (Å): Sb(1)-O(1) 2.0402(13), Sb(1)-O(2) 2.0256(13), Sb(1)-C(30) 2.1328(19), Sb(1)-C(36) 2.1383(19), Sb(1)-C(42) 2.1054(19), O(1)-C(1) 1.358(2), O(2)-C(2) 1.366(2), O(3)-C(19) 1.376(2), O(3)-H(1) 0.73(3), S(1)-C(7) 1.8365(18), S(1)-C(16) 1.7772(19), C(1)-C(2) 1.398(3), C(1)-C(6) 1.393(3), C(2)-C(3) 1.392(3), C(3)-C(4) 1.415(3), C(4)-C(5) 1.400(3), C(5)-C(6) 1.401(3), C(16)-C(17) 1.385(3), C(16)-C(21) 1.389(3), C(17)-C(18) 1.394(3), C(18)-C(19) 1.413(3), C(19)-C(20) 1.408(3), C(20)-C(21) 1.390(3).

The molecular structure of triphenylantimony(V) catecholate **1** in crystal is shown in Figure 2. The central antimony atom Sb(1) has a distorted square pyramidal geometry with an O,O’-chelating ligand and two phenyl groups in the basal plane. The bond angles in the base of pyramid O(1)-Sb(1)-C(30) and O(2)-Sb(1)-C(36) are 156.73(6) and 142.01(7)°. The bond Sb(1)-C(42) with the apical phenyl group is approximately 0.028(2)-0.033(2) Å shorter than the equatorial bonds Sb(1)-C(30) and Sb(1)-C(36). The planes of the aromatic rings C(16-21) and C(1-6) of the phenolic group and the catecholate ligand, respectively, lie at an angle of 57.6(1)º. The arylthio-fragment in **1** is stronger turned away from the catecholate ring than in catechol **L_1_**, the torsion angle C(2)C(3)C(7)S(1) in **1** is 77.9(1)° (vs. 56.7(1)° in **L_1_**), and the torsion angle C(3)C(7)S(1)C(16) is 138.1(1)° (vs. 47.9(1)° in **L_1_**).

The oxygen-to-carbon bonds O(1)-C(1) and O(2)-C(2) (1.358(2) and 1.366(2) Å, respectively) are ordinary and typical for catecholato complexes of different metals [65], and bond O(3)-C(19) (1.376(2) Å) is the longest O-C bond in **1**, which is typical for sterically hindered phenols [66,67,68]. The six-membered carbon ring C(1-6) is aromatic, with average C-C bond distances of 1.400 ± 0.015 Å, which is very close to the same value for catechol L_1_. In general, the geometrical characteristics of the redox-active catecholato ligand in **1** are typical for various antimony(V) catecholates [69,70,71,72,73,74,75,76,77,78,79].

In the crystal, molecules **1** form layers, where one can find intermolecular hydrogen bonding between the phenol group of one complex molecule and the sulfur atom of a neighboring complex molecule (Figure S10) with a distance O(3)-H(1)…S(1) of 2.86(1) Å and corresponding angle O(3)-H(1)-S(1) of 115.5(1)°.

### 2.3. Electrochemistry

The electrochemical properties of catechol thioethers **L_1_-L_3_** and triphenylantimony(V) catecholate complexes **1**, **2** were investigated by cyclic voltammetry (CV) in CH_2_Cl_2_ and MeCN solutions containing 0.15 M NBu_4_ClO_4_ (TBAP) as the supporting electrolyte at a glassy carbon working electrode. The redox potentials given in Table 1; Table 2 are referenced to Ag/AgCl/KCl(sat.) electrode.

**Figure 3:**
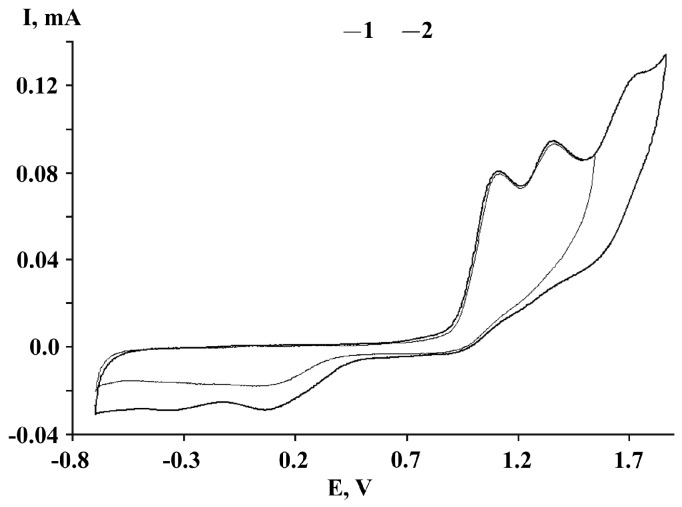
Cyclic voltammograms of compound **L_1_** (in the potential switch from −0.70 to 1.5 V—curve 1; in the potential switch from −0.70 to 1.85 V—curve 2) (MeCN, C = 3 mM, 0.15 M TBAP, scan rate 200 mV∙s^−1^).

**Scheme 3:**
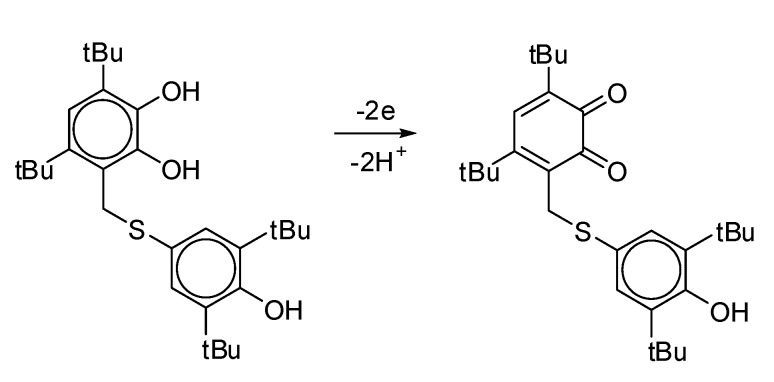
The first stage of electrochemical oxidation of **L_1_**.

Compounds **L_1_-L_3_** are characterized by the presence of multiple electroactive fragments: catechol, phenolic group, and thioether linker, which affects their electrochemical behavior. The electrooxidation of the target catechol thioethers occurs in three or two successive stages depending on the applied solvent. Figure 3 shows the cyclic voltammograms (CVs) of **L**_1_ in acetonitrile solution. The CV of **L_1_** in dichloromethane is shown in Figure S11 of ESI. The first two-electron electrochemical stage is ascribed to the irreversible oxidation of the catechol fragment in the case of compounds **L_1_** and **L_3_**. A similar behavior is typical for substituted catechols in aprotic solvents: 3,5-di-*tert*-butylcatechol has an irreversible oxidation peak at 1.11 (CH_3_CN) or 1.23 V (CH_2_Cl_2_). In the first stage, the mechanism of electooxidation involves electron transfer followed by fast deprotonation, leading to *o*-quinone formation (Scheme 3).

Earlier spectroelectrochemical investigations of catechol **L_3_** have shown that electrolysis at the controlled potential leads to the formation of the corresponding *o*-benzoquinone [48]. The formation of the *o*-quinone structure is accompanied by the appearance of a wide absorption band at 400–500 nm. A wide absorption band in the visible spectral range is a characteristic feature of compounds containing a quinoid fragment [80]. The spectroelectrochemistry of **L_1_** under the controlled potential electrolysis also confirms the formation of the corresponding *o*-benzoquinone.

**Figure 4:**
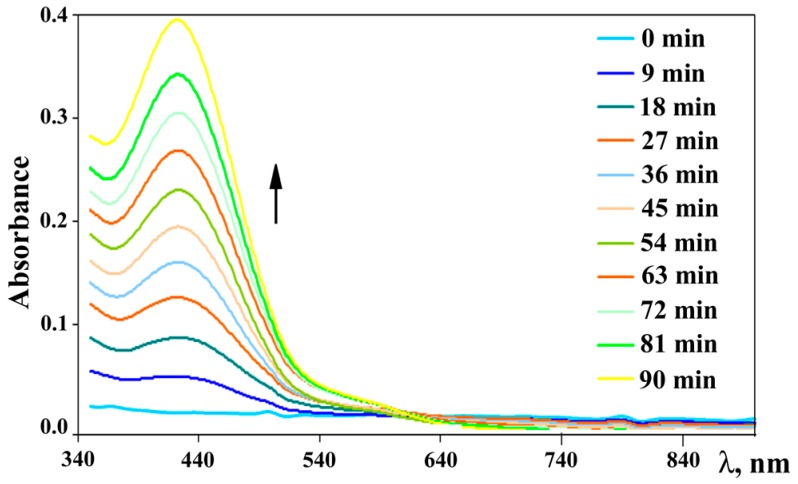
The changes in the absorption spectrum of thioether **L_1_** during the electrolysis time (90 min) at a controlled potential 1.2 V (MeCN, Ar, C = 0.5 mM, 0.15 M TBAP).

As a result of electrooxidation, new absorption with a maximum at 410 nm was observed in the visible region of the spectrum and the intensity of this band increased with time (Figure 4). Additionaly, the second characteristic band of lower intensity in the form of a shoulder to the main band appears in the region of 590 nm, which corresponds to the n-π * transition in quinones.

The absorption band in the visible spectral range corresponds to a π-π* transition, which is a characteristic for compounds containing a quinonoid fragment like 3.5-di-*tert*-butyl-*o*-benzoquinone [81]. The CV of **L_1_** after electrolysis in the cathodic region contains a quasi-reversible peak at E_1/2_ = −0.58 V (Figure S12 of ESI). This peak is assigned to the reduction of electrogenerated *o*-benzoquinone to *o*-benzosemiquinone.

The second oxidation peak on CVs of catechols **L_1_**, **L_3_** may be assigned to the oxidation of the phenolic group. In acetonitrile, for compounds **L_1_** and **L_3_**, the shift of the second oxidation potential to the cathode region occurs as compared to the oxidation of the well-known phenolic antioxidant (ionol)—2,6-di-*tert*-butyl-4-methylphenol 1.47 V (CH_3_CN). In dichloromethane, the oxidation potentials of **L_1_** and **L_3_** are close to the value for this phenolic compound (1.48 V). The presence of the thioether group causes the appearance of the third redox wave at high anodic potentials. For **L_1_** and **L_3_** in acetonitrile, the values E^ox3^ are in good agreement with the data on the oxidation of catechol thioethers: the electrooxidation of the thioether group occurs in the potential range from 1.59 to 1.67 V. On the CVs of **L_1_**, **L_3_** in dichloromethane, two oxidation waves only are observed and they are shifted to the anodic region by 0.15 and 0.19 V in comparison with the data in MeCN. Obviously, the third anode process occurs at the higher anodic potential restricted by the electrochemical oxidation of the solvent.

**Figure 5:**
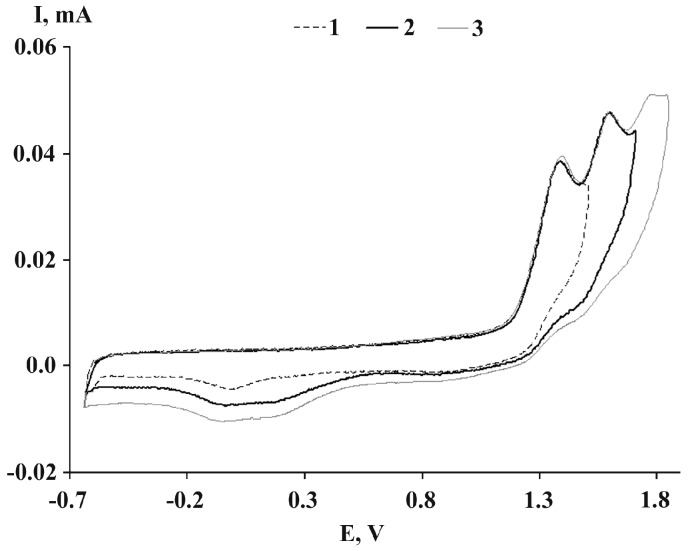
The CV curves of **L_2_** (in the potential switch from −0.60 to 1.50 V—curve 1; in the potential switch from −0.60 to 1.70 V—curve 2; in the potential switch from −0.60 to 1.85 V—curve 3) (CH_2_Cl_2_, C = 1.5 mM, 0.15 M TBAP, scan rate 200 mV∙s^−1^).

The CV of **L_2_** in dichloromethane solution has three oxidation peaks like the CVs of **L_1_** and **L_3_** in acetonitrile (Figure 5).

**Scheme 4:**
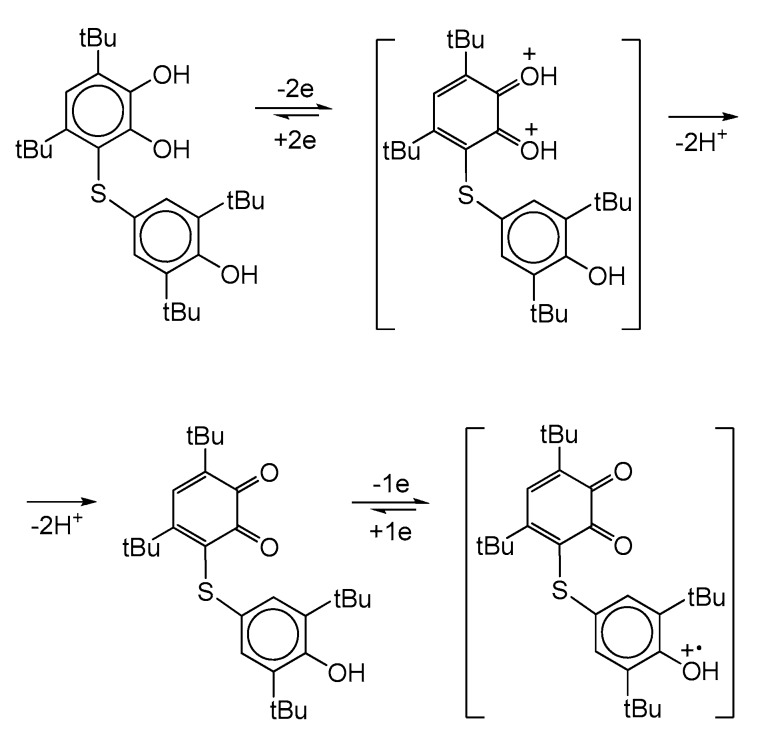
The electrochemical oxidation of **L_2_**.

In contrast to the above described catechol-thioesters **L_1_** and **L_3_**, the first and second oxidation stages for compound **L_2_** are quasi-reversible. The obtained values of the current ratio (I_c_/I_a_) indicate a low stability of the electrically generated intermediates. The introduction of *tert*-butyl groups helps to stabilize the dication formed in the first stage (like in the case of **L_1_**); however, nevertheless, a rapid deprotonation reaction occurs in the solution, and, as a result, a small reduction peak is observed in the reverse scan of CV. At the second stage, one-electron oxidation of a sterically hindered phenolic fragment leads to the formation of a cation-radical intermediate, which is more stable over the time of the CV experiment (Scheme 4).

The third anode peak at the potential of 1.77 V suggests the participation of the thioether fragment in further redox transformations.

It should be noted that the presence of an electron-withdrawing sulfur atom in the catecholate ring or the presence of a methylene bridge between the aromatic ring and the heteroatom does not practically affect the oxidation potentials of compounds **L_1_** and **L_3_** in dichloromethane (Table 1). At the same time, the first oxidation potential (0.05 V) for **L_3_** is shifted to the anode region as compared with 3,5-di-*tert*-butylcatechol in accordance with the electro-acceptor effect of the sulfur atom. In acetonitrile, such changes are less pronounced. In dichloromethane, for compounds **L_2_** and **L_3_**, which differ by the absence or presence of *tert*-butyl groups in the phenolic fragment, a shift of the oxidation potentials (0.11 and 0.10 V) for **L_2_** to the anode region is observed. This behavior can be rationalized by the formation of stronger intramolecular hydrogen bonds between the catechol hydroxyl and the sulfur atom than in the case of compound **L_1_**. Using S-functionalized phenols as an example, such an interaction led to an increase in the bond-breaking energy, BDE (O-H) [82]. The 4-hydroxyphetylthio group introduced into the catecholate cycle exhibits an electron-withdrawing effect, which is expressed for **L_2_** in a significant shift (0.14 V) of the first oxidation potential to the anode region as compared with 3,5-di-*tert*-butylcatechol.

**Scheme 5:**
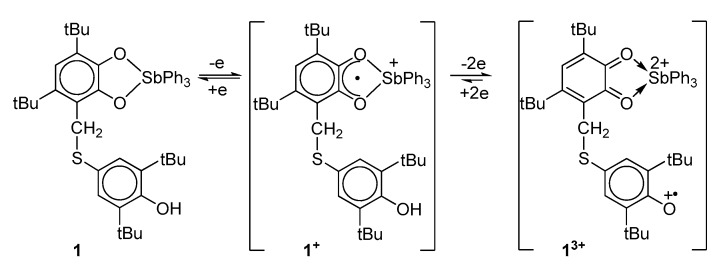
The electrochemical oxidation of complex **1**.

As in the case of free ligands, the solvent has a significant effect on the electrochemical behavior of complexes **1** and **2**. In dichloromethane, for both complexes, the first stage of oxidation has a quasi-reversible one-electron character and leads to the formation of relatively stable (in the CV time scale) monocationic complexes of the type [(SQ)SbPh_3_]^+^ (Scheme 5) [83,84]. The value of the E^ox1^_1/2_ potential for **1** is similar to the data obtained for triphenylantimony(V) 3,6-di-*tert*-butylcatecholate [85]. For the complex **2**, this value shifts by 0.06 V to the anode region, which is associated with the electron-withdrawing effect of the sulfur atom, which is not separated from the catecholate ring by the methylene group as in case of **1**. Such changes are in good agreement with previously obtained results for triarylantimony(V) 6-chloro(bromo)-3,5-di-*tert*-butylcatecholates [86,87]. The introduction of a methylene group between the catecholate fragment and the 3,5-di-*tert*-butyl-4-hydroxyphenylthio group has no practically effect on the value of the E^ox1^_1/2_ potential, as in the case of 6-alkoxymethyl-substituted 3,5-di-*tert*-butylcatecholates of triphenylantimony(V) [88].

**Figure 6:**
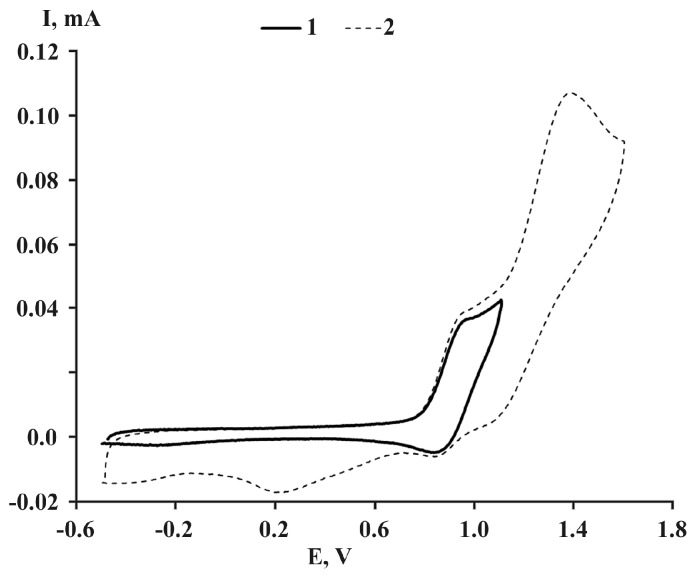
Cyclic voltammograms of compound **1** (in the potential switch from −0.5 to 1.1 V—curve 1; in the potential switch from −0.5 to 1.6 V—curve 2) (CH_2_Cl_2_, C = 3 mM, 0.15 M TBAP, scan rate 200 mV∙s^−1^).

For complex **1**, the second anode peak has a quasi-reversible character (Figure 6). A double increase in the current of the second oxidation peak as compared to the first one indicates an increase in the number of electrons involved in this electrode process to two.

The value of the peak potential E^ox2^_p_ = 1.39 V on the CV of **1** is fixed in the potential range of 1.37–1.60 V, which is characteristic of the second redox process “*o*-semiquinone/*o*-benzoquinone” in such triarylantimony(V) catecholates [89,90,91]. This electrochemical picture in the case of **1** indicates the convergence of the boundary redox orbitals of *o*-semiquinone and phenolic fragments in **1^+^**, which leads to their simultaneous electrooxidation. A decrease in the ratio of currents to 0.5 for the second oxidation process is caused by the subsequent chemical stages in the solution—deprotonation of the phenoxyl radical cation and decoordination of *o*-benzoquinone moiety from the tricationic intermediate.

**Scheme 6:**
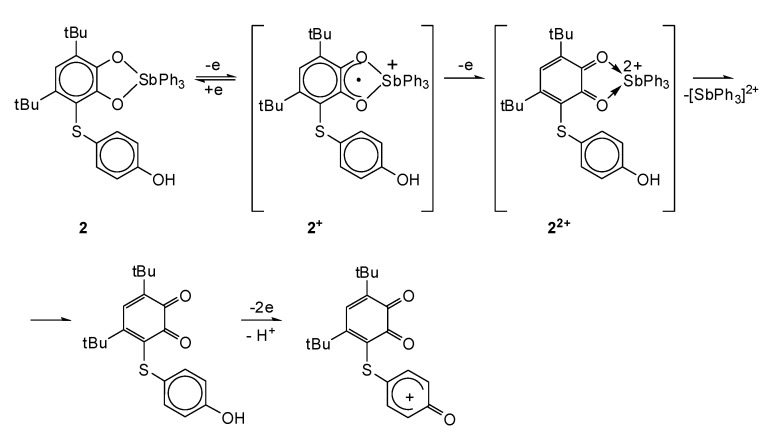
The electrochemical oxidation of complex **2**.

**Figure 7:**
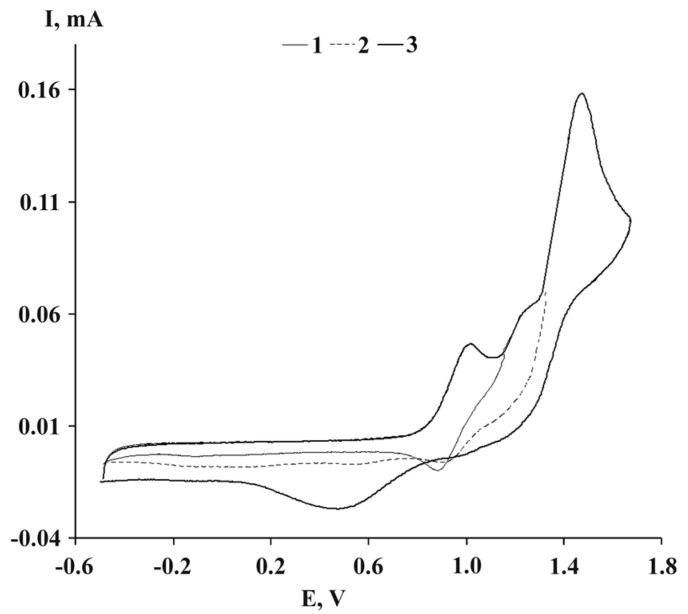
Cyclic voltammograms of compound **2** (in the potential switch from −0.50 to 1.15 V—curve 1; in the potential switch from −0.50 to 1.30 V—curve 2; in the potential switch from −0.50 to 1.70 V—curve 3) (CH_2_Cl_2_, C = 3 mM, 0.15 M TBAP, scan rate 200 mV∙s^−1^).

In complex **2**, these redox centers behave separately: The second oxidation stage at 1.24 V is irreversible and assigned to the further oxidation of coordinated *o*-benzosemiquinone in the intermediate [(SQ)SbPh_3_]^+^ (**2^+^**) to *o*-benzoquinone, with the subsequent decoordination of *o*-benzoquinone from **2^2+^** leading to the complex decomposition (Scheme 6). The third redox process is a two-electronic peak, which is an oxidation of the phenolic group in free *o*-benzoquinone at 1.48 V (Figure 7).

The potential value of this third redox-stage is identical with that one for the oxidation of the phenolic group in free **L_3_** and 2,6-di-*tert*-butyl-4-methylphenol (1.48 V vs. Ag/AgCl) [92].

In a coordinating solvent acetonitrile (Table 2), the oxidation potentials for complexes **1** and **2**, as it was expected, shift slightly to the cathode region. In the whole, the electrochemical behavior of the complexes changes. The CV of **1** in acetonitrile has three oxidation waves (Figure S13).

**Scheme 7:**
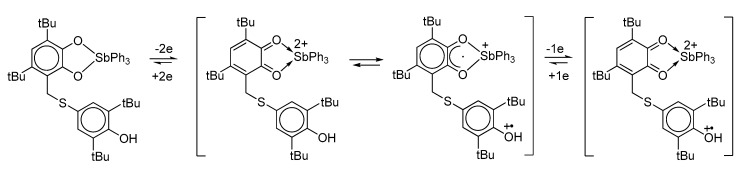
The electrochemical oxidation of complex **1** in acetonitrile.

In acetonitrile, the current of the first quasi-reversible oxidation peak for **1** increases twice to a two-electron level, and the ratio of currents I_c_/I_a_ decreases in comparison with the data obtained for **1** in dichloromethane. These changes indicate the generation of an unstable dicationic intermediate [(Q)SbPh_3_]^2+^ in the near-electrode region. Earlier, we observed a similar electrochemical activity for the triphenylantimony(V) catecholates containing electron-withdrawing groups in the catecholate cycle, but in most cases, the dicationic derivatives formed in the electrooxidation were unstable [90,91]. The second wide quasi-reversible oxidation peak at 1.24 V is single electron in nature. The low value of the current ratio, as in the first case, indicates the instability of the resulting intermediate and the course of the subsequent chemical stage. The proximity of the redox orbitals of the catecholate ligand and the sterically hindered phenol fragment does not allow us to unambiguously describe the oxidative transformations at the first and second stages of oxidation. Based on the value of the first oxidation potential, we may assume that the dianionic catecholate fragment is oxidized initially to *o*-benzoquinone, accompanied by an electron transfer from the phenolic group to the dication (Scheme 7). The second anode process may equally be attributed to the oxidation of a coordinated *o*-semiquinone radical anion to *o*-benzoquinone or to the oxidation of a di-*tert*-butylphenol moiety to a radical cation.

**Figure 8:**
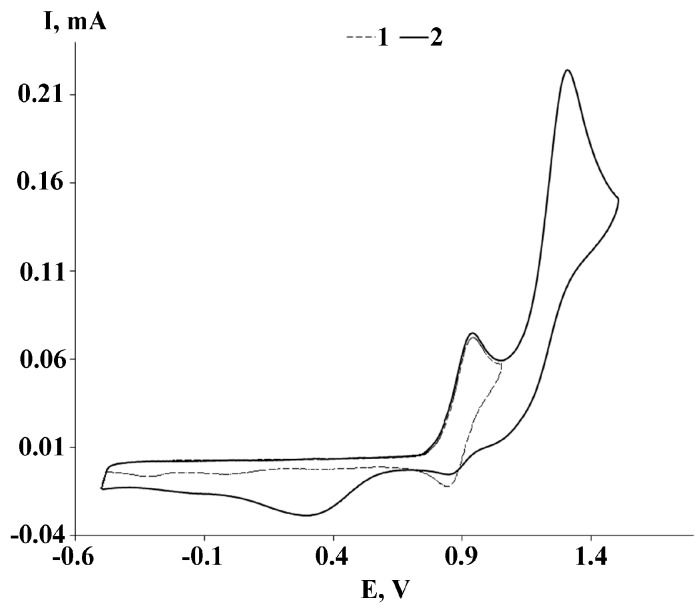
Cyclic voltammograms of compound **2** (in the potential switch from −0.50 to 1.05 V—curve 1; in the potential switch from −0.50 to 1.50 V—curve 2) (CH_3_CN, C = 4 mM, 0.15 M TBAP, scan rate 200 mV∙s^−1^).

The electrooxidation of **2** in acetonitrile (Figure 8) has a quasi-reversible one-electron character at the first stage (as in dichloromethane), which indicates the generation of a monocation complex containing a coordinated *o*-semiquinone ligand.

A significant difference of the electrochemical behavior of **2** in acetonitrile from the electrochemical picture of **2** in dichloromethane is the absence of a separation of the second and third stages of oxidation: There is one two-electron oxidation peak. Complex **2** in acetonitrile exhibits a behavior similar to **1** in dichloromethane: Both redox active fragments of the ligand are involved in the second stage of oxidation, namely the *o*-semiquinone ligand is converted to *o*-benzoquinone, and the phenolic group is oxidized to the radical cation.

The synthesized ligands (as well as the complexes based on them) contain several electroactive centers. Complexes **1** and **2** significantly expand their redox capabilities owing to the fact that they can act as: (1) electron donors due to the catecholate metallocycle capable of sequential oxidations, and (2) donors of the hydrogen atoms forming a stable phenoxyl radical. This ability of ligands can be used in catalysis in coordination with transition metal ions.

In contrast to the previously studied triphenylantimony(V) catecholato complexes with additional redox centers (ferrocenyl, morpholine, piperazine groups [18,24,30]), whose electrochemical activation is observed at potentials shifted to the cathode region as compared to the “catechol/*o*-semiquinone” transition, compounds **1** and **2** are characterized by electrooxidation of the phenolic fragment at potentials that are close to the second redox transition “*o*-semiquinone/*o*-benzoquinone”. This fact indicates the convergence of the boundary redox orbitals of the phenolic and *o*-semiquinone fragments in the electro-generated monocationic complexes.

### 2.4. EPR Experiments

The oxidation of the catechol thioether **L_1_** with lead dioxide in toluene proceeds slowly. In this case, immediately after mixing the reagents, a superposition of two doublets was observed (Figure S14(1)). One of the doublets has the following parameters: g_i_ = 2.0020, a_i_(^1^H) = 3.6 G, the second: g_i_ = 2.0009, a_i_(^1^H) = 3.85 G. The intensive stirring and heating of the reaction mixture leads to an increase in the intensity of the EPR spectrum, and the intensity of the second doublet grows significantly more noticeably in comparison with the intensity of the first doublet (Figure S14(2)). An increase in the signal intensity allows one to see the satellite splitting of the second signal on magnetic isotopes of lead (^207^Pb, I = 1/2, 22.1% [93]) with the hyper-fine structure (HFS) constant a_i_(^207^Pb) = 70.5 G. The doublet with satellite splitting was simulated using the WinEPR SimFonia 1.25 program, and the simulation spectrum is shown in Figure S14(3).

**Scheme 8:**
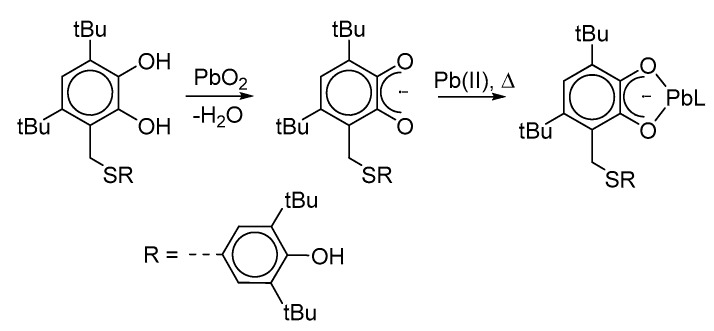
The oxidation of catechol **L_1_** with lead(IV) oxide.

It can be concluded that the interaction of catechol **L_1_** with lead dioxide slowly leads to the deprotonation of catechol with the formation of the *o*-semiquinone radical anion at the first stage (Scheme 8), which has a doublet with parameters g_i_ = 2.0020, a_i_(^1^H) = 3.6 G without any satellite splitting on the lead isotopes.

The process accelerates when the mixture is heated; however, the formation of *o*-semiquinone complex with lead(II) occurs, the EPR spectrum of which increases in intensity with the heating of the sample (the second EPR spectrum).

**Figure 9:**
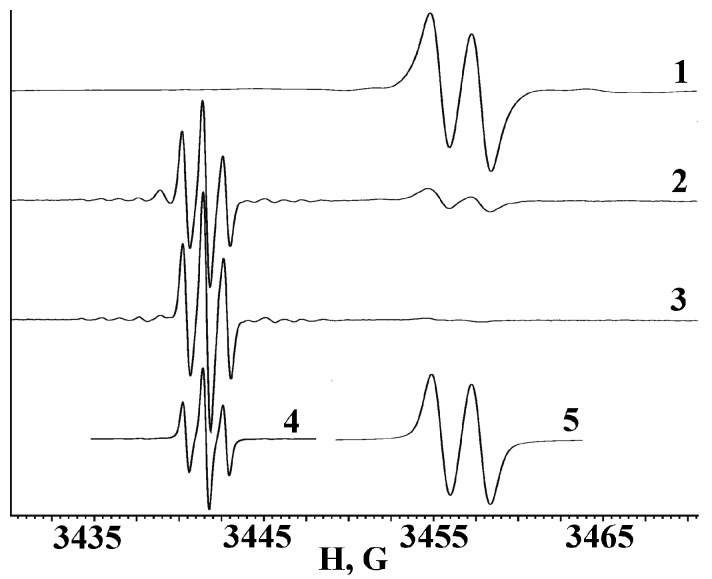
The X-band EPR spectrum of the mixture “**L_2_** + PbO_2_” in toluene immediately after mixing the reagents (spectrum 1), after stirring for 10 min (spectrum 2), and after stirring for 20 min (spectrum 3); Simulated EPR spectra (WinEPR SimFonia 1.25): triplet with parameters g_i_ = 2.0050, a_i_(2^1^H) = 1.1 G (spectrum 4), doublet with parameters g_i_ = 1.9967, a_i_(^1^H) = 2.3 G (spectrum 5).

The oxidation of catechol **L_2_** with lead dioxide in a toluene solution proceeds more easily than the oxidation of **L_1_** and does not require heating. In this case, initially (Figure 9(1)), a wide doublet appears with the parameters g_i_ = 1.9967, a_i_(^1^H) = 2.3 G, which disappears gradually with further stirring of the reaction mixture (Figure 9(2)) with the simultaneous appearance of a triplet with some other parameters (g_i_ = 2.0050, a_i_(2^1^H) = 1.1 G). Twenty minutes after the beginning reaction, only one triplet signal is observed practically (Figure 9(3)). Both spectra were simulated using WinEPR SimFonia 1.25. Simulations of the triplet and doublet spectra are shown in Figure 9(4),(5), respectively.

**Scheme 9:**
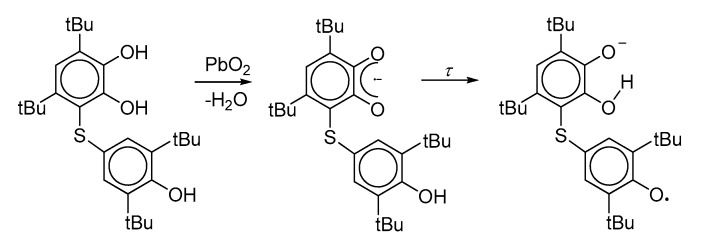
The oxidation of **L_2_** with lead(IV) oxide.

The oxidation of **L_2_** with lead dioxide proceeds with the deprotonation of catechol with the formation of the *o*-semiquinone radical (Scheme 9), and the doublet signal from this radical is recorded at the initial time. However, a decrease in the intensity of this signal and an increase in the intensity of the triplet with the HFS constant on two equivalent protons of 1.1 G characteristic of 4,6-di-*tert*-butyl-phenoxyl radicals gradually occurs. Thus, we can conclude that either the *o*-semiquinone formed at the initial stage oxidizes and deprotonates the phenolic group to form a phenoxyl radical during the reaction, or (since the reaction with lead dioxide is heterogeneous, requiring an excess of oxidizing agent) during the reaction, oxidation of the 4,6-di-*tert*-butylphenol group in the catechol proceeds more slowly than the oxidation of the catechol fragment, and at a certain moment, the concentration of the *o*-semiquinone radical begins to decrease with a simultaneous increase in the concentration of the phenoxyl radical.

Earlier, it was shown that thioether **L_3_** is oxidized by lead(IV) oxide in toluene solution with the formation of lead *o*-semiquinonato derivatives [14a]. In the EPR spectrum, a doublet at g_i_ = 2.0004 with satellite splitting, typical for lead(II) *o*-semiquinolates, was observed. This fact is also explained by the initial formation of the *o*-semiquinone radical anion further coordinated to metal. The hyperfine structure of the EPR spectrum of a system “**L_3_**+PbO_2_” is caused by hyperfine splitting of a signal on the one proton nucleus in the fifth position of the *o*-semiquinone six-membered carbon ring with a satellite splitting on the magnetic lead isotope (^207^Pb, 22.1%, I = 1/2): a_i_(^1^H) = 2.65 G, a_i_(^207^Pb) = 22.75 G [48].

**Scheme 10:**
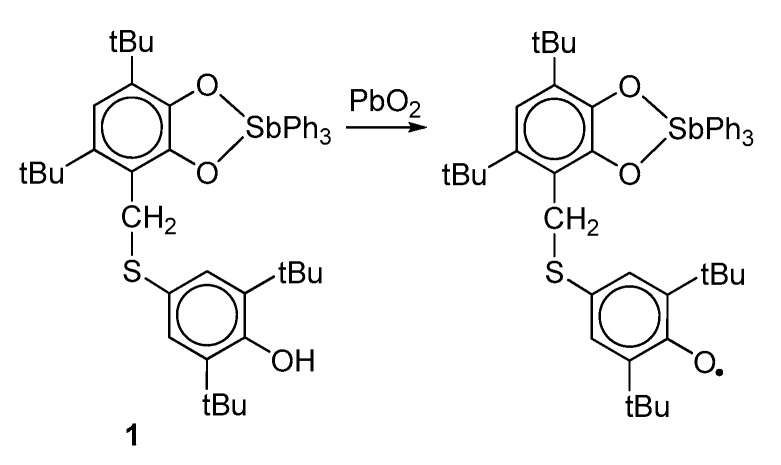
The oxidation of complex **1** with PbO_2_ with formation of the phenoxyl radical.

**Figure 10:**
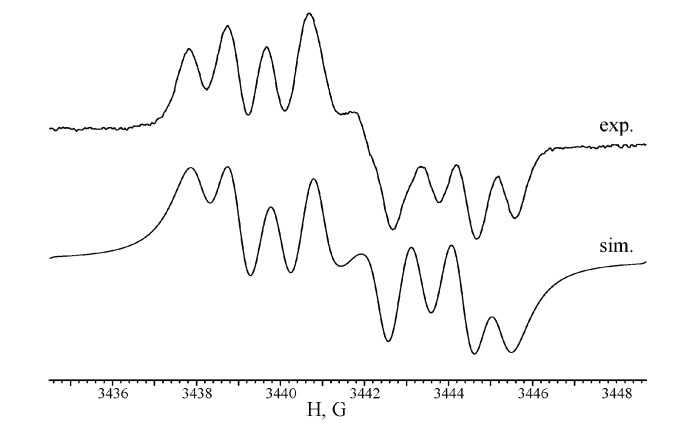
The X-band EPR spectrum of a mixture ”**1**+PbO_2_”: exp.—the experimental spectrum (toluene, 298 K); sim.—its simulation (WinEPR SimFonia 1.25).

The oxidation of triphenylantimony(V) catecholate **1** with lead(IV) oxide in toluene proceeds with weak heating (to 50°C) and leads to the formation of the corresponding phenoxyl radical (Scheme 10) with an isotropic multiplet EPR spectrum (Figure 10) with g_i_ = 2.0050. The spectrum was simulated with Easyspin 5.2.25 [94], and the following HFS constants were revealed: the hyperfine coupling with two aromatic protons in meta-positions to the oxy group with a_i_(2 ^1^H) = 0.89 G, and coupling with two non-equivalent protons of the S-CH_2_ group in the para-position of the phenoxyl ring with a_i_(1 ^1^H) = 1.97 G and a_i_(1 ^1^H) = 3.30 G. The nonequivalence of these methylene protons is caused by their different positions towards the pi-system of the phenoxyl radical.

The EPR data of experiments on the oxidation of free ligands indicate the tendency of **L_1_** and **L_2_** to form *o*-semiquinone radical anions. The difference is the great propensity of **L_1_** to form stable chelate rings, while for **L_2_** the subsequent generation of a stable phenoxyl radical is possible. Complex **1** in reaction with lead(IV) oxide tends to form a phenoxyl radical, which is consistent with the possibility of simultaneous activation of the catecholate cycle and the phenolic group during electrooxidation in a polar solvent (MeCN).

## 3. Materials and Methods

### 3.1. General Remarks

All the experiments on the synthesis and study of properties of complexes were carried out in evacuated ampoules in the absence of oxygen and water. The solvents used were purified and dried by standard methods [95]. The infrared spectra of the complexes in the 4000-400 cm^−1^ range were recorded on an FSM 1201 Fourier-IR spectrometer in nujol mull. The NMR spectra of **L_1_** and **L_2_** were recorded in CDCl_3_ solution using a “Bruker DPX 200” instrument (200 MHz for ^1^H, and ~50 MHz for ^13^C), and the NMR spectra of **1** and **2** were recorded in CDCl_3_ solution using a “Bruker ARX 400” instrument (400 MHz for ^1^H, and ~100 MHz for ^13^C), with Me_4_Si as the internal standard. The C, H, S elemental analysis was performed on an Elemental Analyzer “Elementar vario EL Cube”; the antimony content was accomplished by combustion analysis.

### 3.2. Cyclic Voltammetry

The electrochemical studies were carried out using IPC-Pro potentiostate in three-electrode mode. The stationary glassy carbon (d = 2 mm) disk was used as the working electrode; the auxiliary electrode was a platinum-flag electrode. The reference electrode was Ag/AgCl/KCl(sat.) with a watertight diaphragm. All measurements were carried out under argon. The samples were dissolved in the pre-deaerated solvent. The rate scan was 0.2 Vs^−1^. The supporting electrolyte 0.15 M [Bu_4_N]ClO_4_ (99%, «Acros») underwent recrystallization twice from aqueous EtOH and then it was dried in vacuum (48 h) under 50°C. The concentration of the compounds was 1.5–4.0 mM.

### 3.3. X-Ray Diffraction Studies

The X-ray diffraction data were collected on a SMART APEX I (**L_1_**, **1**) diffractometer (graphite monochromated, Mo_Kα_-radiation, ω-scan technique, λ = 0.71073 Å) at 100 K. The intensity data were integrated by the SAINT program [96]. SADABS [97] was used to perform area-detector scaling and absorption corrections. The structures were solved by direct methods and were refined on F^2^ using the SHELXTL package [98]. All non-hydrogen atoms were refined anisotropically. All hydrogen atoms were placed in geometrically idealized positions and treated as riding with U_iso_(H) = 1.2 Ueq (U_iso_(H) = 1.5 Ueq for the hydrogen atoms in CH_3_ groups) of their parent atoms. Crystal data and details of the data collection and structure refinement for **L_1_** and **1** are given in Table S1. The selected bond lengths for **L_1_** are listed in Table S2, and the selected bond angles for **1** are in Table S3 of ESI. The structure parameters were deposited with the Cambridge Structural Database (CCDC 1978538 (**L_1_**) and 1978539 (**1**); deposit@ccdc.cam.ac.uk or http://www.ccdc.cam.ac.uk/data_request/cif).

### 3.4. Synthesis of Catechol Thioethers

3,5-Di-*tert*-butyl-6-methoxymethylcatechol and 4,6-di-*tert*-butyl-3-(4-hydroxyphenylthio)catechol (**L_3_**) were synthesized according to previous reports [8c,14a].

#### 3.4.1. 4,6-Di-tert-butyl-3-(3,5-di-tert-butyl-4-hydroxyphenyl-thiomethyl)catechol L1

Ligand was prepared according the next procedure: 3,5-di-*tert*-butyl-6-methoxymethylcatechol (1.5 mmol, 0.400 g) and 2,6-di-*tert*-butyl-4-mercaptophenol (1.5 mmol, 0.357 g) were dissolved in acetic acid (15 mL) under argon atmosphere, then the mixture was heated for 12 h at 60 °C. Further water (25 mL) was added to the reaction mixture and a precipitate was filtrated. The product was dried under vacuum, then it was recrystallized from n-hexane to form white crystals. The yield was 0.37 g (52%). M.p. 174–176 °C. Anal. calc. for C_29_H_44_O_3_S (%): C, 73.68; H, 9.38; S, 6.78; found: C, 73.54; H, 9.42; S, 6.70. ^1^H NMR (200 MHz, CDCl_3_, δ, ppm): 1.22 (s., 9 H, tBu, Cat), 1.37 (s., 18 H, tBu, Phenol), 1.42 (s., 9 H, tBu, Cat), 4.35 (s., 2 H, CH_2_-S), 5.30 (s., 1 H, OH), 6.12 (s., 1 H, OH), 6.89 (s., 1 H, arom. C_6_H_1_), 7.18 (s., 2 H, arom. C_6_H_2_), 7.26 (s., 1 H, OH). ^13^C{^1^H} NMR (50 MHz, CDCl_3_, δ, ppm): 29.48, 30.07, 31.99, 34.27, 34.93, 35.43, 36.40, 116.73, 119.28, 121.59, 130.95, 133.64, 136.78, 138.71, 142.91, 143.34, 154.59. FT-IR (KBr): ν = 3629, 3510, 3198, 3057, 2955, 2910, 2870, 1481, 1425, 1370, 1290, 1235, 1144 cm^−1^.

#### 3.4.2. 4,6-Di-*tert*-butyl-3-(3,5-di-*tert*-butyl-4-hydroxyphenyl-thio)catechol **L_2_**

This ligand was prepared according to the followed protocol. 2,6-Di-*tert*-butyl-4-mercaptophenol (1 mmol, 0.238 g) in 10 mL ethanol was added dropwise to a solution of 3,5-di-*tert*-butyl-*o*-benzoquione in 20 mL of ethanol (0.5 mmol, 0.110 g) over a period of 2–3 h and the mixture was stirred under argon till decoloration of the reaction media at room temperature. The volume was concentrated under a reduced pressure to yield a crude solid. The product was recrystallized from dry ethanol, dried under vacuum, and isolated as yellow powder. The yield was 0.08 g (35%). M.p. 165–167 °C. Anal. calc. for C_28_H_42_O_3_S (%): C, 73.32; H, 9.23; S, 6.99; found: C, 73.48; H, 9.26; S, 6.87. ^1^H NMR (200 MHz, CDCl_3_, δ, ppm): 1.25 (s., 18H, tBu), 1.31 (s., 9H, tBu), 1.41 (s., 9H, tBu), 4.93 (s., 1H, OH), 6.87 (s., 1H, C_6_H_1_), 6.91 (s, 2H, C_6_H_2_). ^13^C{^1^H} NMR (50 MHz, CDCl_3_, δ, ppm): 29.35, 29.63, 34.90, 34.98, 35.02, 118.05, 119.13, 127.73, 134.06, 136.00, 136.29, 138.40, 143.23, 146.14. FT-IR (KBr): ν = 3629, 3485, 2958, 2908, 2868, 1644, 1663, 1485, 1458, 1392, 1363, 1218, 1164 cm^−1^.

### 3.5. Synthesis of Complexes

#### 3.5.1. Complex (6-(CH_2_-S-tBu_2_Phenol)-3,5-DBCat)SbPh_3_ (**1**)

A solution of 4,6-di-*tert*-butyl-3-(3,5-di-*tert*-butyl-4-hydroxyphenyl-thiomethyl)catechol **L_1_** (0.236 g, 0.5 mmol) in 25 mL of toluene was added with stirring to a toluene solution of triphenylantimony dibromide (0.256 g, 0.5 mmol, 10–15 mL of toluene) under argon atmosphere. Then two equivalents of the triethylamine (0.14 mL, 1 mmol) were added to the toluene solution. After the addition of reagents and changing the color of the solution by yellow to orange, the reaction mixture was stirred for 2 h. The formed white precipitate of triethylammonium bromide was filtered off. The volume of filtrate was concentrated under a reduced pressure to a half volume and stored at −18°C for five days. The X-ray-suitable yellow crystals of **1** were collected by decantation, and dried under vacuum. The yield was 0.300 g (73%). Anal. calc. for C_47_H_57_O_3_SSb (%): C, 68.53; H, 6.97; S, 3.89; Sb, 14.78. Found (%): C, 68.62; H, 7.05; S, 4.01; Sb, 14.53. ^1^H NMR (400 MHz, δ, ppm): 1.40 (s, 18H, tBu), 1.41 (s, 9H, tBu), 1.42 (s, 9H, tBu), 4.55 (s, 2H, CH_2_S), 5.18 (s, 1H, OH), 6.70 (s, 1H, arom. C_6_H_1_), 7.36 (s, 2H, arom. C_6_H_2_), 7.40–7.50 (m, 9H, Ph), 7.87–7.91 (m, 6H, Ph). ^13^C{^1^H} NMR (100 MHz, δ, ppm): 29.63, 30.19, 32.43, 34.36, 34.65, 35.85, 36.48, 112.61, 117.58, 127.81, 128.75, 129.06, 131.02, 131.66, 135.44, 136.27, 136.75, 137.81, 142.56, 147.52, 152.86. FT-IR (KBr): ν = 3541, 3068, 3045, 2957, 2910, 2873, 1580, 1480, 1431, 1394, 1360, 1258, 1240, 1168 cm^−1^.

#### 3.5.2. Complex (6-(S-Phenol)-3,5-DBCat)SbPh_3_ (**2**)

A solution of the 4,6-di-*tert*-butyl-3-(4-hydroxyphenylthio)catechol (0.173 g, 0.5 mmol) in 20 mL of toluene was added dropwise with stirring to a toluene solution of triphenylantimony dibromide (0.256 g, 0.5 mmol, 10–15 mL of toluene) under argon atmosphere. Then two equivalents of the triethylamine (0.14 mL, 1 mmol) were added to toluene solution. After the addition of reagents and changing the color of the solution to pale yellow, the reaction mixture was stirred for 2 h. The formed white precipitate of ammonium salt was filtered off. The volume was concentrated under a reduced pressure to yield a yellow crude solid. The product was recrystallized from a mixture of the solvents pentane and hexane (v/v = 1/1), dried under vacuum, and isolated as a yellow powder of **2**. The yield was 0.175 g (50%). Anal. calc. for C_38_H_39_O_3_SSb (%): C, 65.43; H, 5.64; S, 4.60; Sb, 17.46. Found (%): C, 65.30; H, 5.72; S, 4.55; Sb, 17.60. ^1^H NMR (400 MHz, δ, ppm): 1.45 (s, 9H, tBu), 1.51 (s, 9H, tBu), 4.51 (br.s, 1H, OH), 6.51 (d.m, J = 8.7 Hz, 2H,C_6_H_4_), 6.82 (s, 1H, Ar), 6.94 (d.m, J = 8.7 Hz, 2H, C_6_H_4_), 7.35–7.41 (m, 6H, Ar), 7.43–7.48 (m, 3H, Ar), 7.51–7.56 (m, 6H, Ar). ^13^C{^1^H} NMR (100 MHz, δ, ppm): 29.53, 31.46, 34.80, 36.69, 112.63, 113.01, 115.48, 127.64, 129.03, 131.00, 131.34, 133.45, 135.10, 137.32, 141.25, 143.19, 149.86, 152.48. FT-IR (KBr): ν = 3411, 3056, 2958, 2910, 2870, 1490, 1431, 1397, 1257, 1238, 1167 cm^−1^.

## 4. Conclusions

Thus, in this work, new polyfunctional sterically hindered catechols with an additional phenolic group connected by a bridging sulfur atom, as well as triphenylantimony(V) catecholates based on these ligands, were synthesized. The molecular structures of free ligand **L_1_** and complex **1** in the crystal state were determined by single-crystal X-ray analysis. The electrochemical investigations have shown that the electrooxidation of ligands in the first stage leads to the formation of the corresponding *o*-benzoquinones. The second anode stage involves the oxidation of the phenolic moiety. The results of the EPR studies confirmed the primary oxidation of the catechol group with the formation of the *o*-semiquinone radical anion. The electrooxidation of complexes **1** and **2** in dichloromethane is somewhat different from each other. For compound **2**, the three oxidation stages are clearly fixed, the first two stages correspond to the conversion of the catecholate form of the ligand to *o*-benzosemiquinone and then to *o*-benzoquinone, and the third peak characterizes the oxidation of the phenolic fragment. For complex **1**, the first redox stage also affects the catecholate group, and the second redox process can involve both *o*-semiquinone and phenolic fragments in this electrode process.

In the coordinating solvent (acetonitrile), the electrochemical behavior of compound **2** becomes identical to that one for complex **1** in dichloromethane. At the same time, in acetonitrile for complex **1**, the current of the first anode peak increases significantly, which implies two-electron oxidation leading to a dication containing the one-electron oxidized form of the catecholate ligand and phenoxyl radical. The oxidation of complex **1** in toluene with lead dioxide leads to the fixation of the phenoxyl radical. This indicates the possibility of activation of the phenolic group during electrooxidation. The combination of electrochemical and spectral data indicates the convergence of the boundary redox orbitals of the phenolic and *o*-semiquinone fragments in the electro-generated monocationic triphenylantimony(V) complexes.

## Figures and Tables

**Table 1:** The CV data for thioethers **L_1_**–**L_3_** and triphenylantimony(V) catecholates **1** and **2** in CH_2_Cl_2_ (GC anode, C = 3·10^−3^ M, Ar, 0.15 M Bu_4_NClO_4_, vs. Ag/AgCl/KCl(sat.)).

Compound	E^ox1^, V ^1^	I_c_/I_a_	E^ox2^, V ^1^	I_c_/I_a_	E^ox3^, V
**L_1_**	1.28	-	1.49	-	-
**L_2_**	1.37 (1.32)	0.4	1.58 (1.55)	0.6	1.77
**L_3_**	1.28	-	1.48	-	-
**1**	0.96 (0.89)	0.8	1.39 (1.27)	0.5	-
**2**	1.04 (0.96)	0.7	1.24	-	1.48

^1^ The half-wave potentials E_1/2_ for quasi-reversible oxidation processes are given in parentheses.

**Table 2:** The CV data for thioethers **L_1_**–**L_3_** and triphenylantimony(V) catecholates **1** and **2** in CH_3_CN (GC anode, C = 3·10^−3^ M, Ar, 0.15 M Bu_4_NClO_4_, vs. Ag/AgCl/KCl(sat.)).

Compound	E^ox1^, V ^1^	I_c_/I_a_	E^ox2^, V ^1^	I_c_/I_a_	E^ox3^, V
**L_1_**	1.09	-	1.34	-	1.69
L_2_ *	1.27	-	1.48	0.3	-
L_3_ **	1.13	-	1.26	-	1.61
**1**	0.89 (0.84)	0.4	1.23 (1.12)	0.5	1.68
**2**	0.94 (0.89)	0.8	1.36	-	-

^1^ The half-wave potentials E_1/2_ for quasi-reversible oxidation processes are given in parentheses. * Data have been received in a mixture “CH_3_CN/ CH_2_Cl_2_” (2:1). ** Data from ref. [48].

## Supplementary Materials

The following are available online at https://www.mdpi.com/1420-3049/25/8/1770/s1, Figures S1–S8: The NMR spectra of compounds, Table S1: Crystal data and structure refinement for L1 and 1, Table S2: The selected bond lengths for L1, Table S3: The selected bond lengths for 1, Figure S9: The intermolecular hydrogen bonds in crystals of L1, Figure S10: The order of complex 1 molecules in crystal cell with the indication of intermolecular hydrogen bonding, Figure S11: The CVs of compound L1 (CH2Cl2), Figure S12: the CV of the electrolysis products of compound L1, Figure S13: The CVs of compound 1 (CH3CN), Figure S14: The X-band EPR spectrum of the mixture “L1 + PbO2”. CCDC 1978538 (L1) and 1978539 (1) contain the supplementary crystallographic data for this paper. These data can be obtained free of charge from The Cambridge Crystallographic Data Centre via www.ccdc.cam.ac.uk/data_request/cif.
