# Supplementary material for: Polyfunctional Sterically Hindered Catechols with Additional Phenolic Group and Their Triphenylantimony(V) Catecholates: Synthesis, Structure, and Redox Properties
Source: Molecules. 2020 Apr 12;25(8):1770. doi: 10.3390/molecules25081770 (PMC7221534; doi:10.3390/molecules25081770)
Supplement: Supplementary file 1 [file molecules-25-01770-s001.pdf]

Supplementary materials

Polyfunctional sterically hindered catechols with additional phenolic group and their triphenylantimony(V) catecholates: Synthesis, structure and redox properties

Ivan V. Smolyaninov[1,2], Andrey I. Poddel'sky[3,*], Susanna A. Smolyaninova[2], Maxim V. Arsenyev[3], Georgy K. Fukin[3], Nadezhda T. Berberova[2]

[1] Federal State Budgetary Institution of Science "Federal research centre The Southern Scientific Centre of the Russian Academy of The Sciences", Toxicology Research Group, Tatischeva str. 16, 414056 Astrakhan, Russian Federation; ivsmolyaninov@gmail.com (I.S.)

[2] Astrakhan State Technical University, Department of Chemistry, 16 Tatisheva str., Astrakhan 414056, Russian Federation; zeynalovasa@mail.ru (S.S.); nberberova@gmail.com (N.B.)

[3] G.A. Razuvaev Institute of Organometallic Chemistry, Russian Academy of Sciences, 49 Tropinina str., 603137 Nizhny Novgorod, Russian Federation; aip@iomc.ras.ru (A.P.); mars@iomc.ras.ru (M.A.); gera@iomc.ras.ru (G.F.)

* Correspondence: aip@iomc.ras.ru; Tel.(Fax): +7-831- 462-7497

Content

Figure S1. The ^{1}H NMR spectrum of $\mathbf{L_1}$ ($CDCl_3$, 200 MHz) 2

Figure S2. The $^{13}C\{^{1}H\}$ NMR spectrum of $\mathbf{L_1}$ ($CDCl_3$, 50 MHz) 2

Figure S3. The ^{1}H NMR spectrum of $\mathbf{L_2}$ ($CDCl_3$, 200 MHz) 3

Figure S4. The $^{13}C\{^{1}H\}$ NMR spectrum of $\mathbf{L_2}$ ($CDCl_3$, 50 MHz) 3

Figure S5. The ^{1}H NMR spectrum of **1** ($CDCl_3$, 400 MHz) 4

Figure S6. The $^{13}C\{^{1}H\}$ NMR spectrum of **1** ($CDCl_3$, 100 MHz) 4

Figure S7. The ^{1}H NMR spectrum of **2** ($CDCl_3$, 400 MHz). 5

Figure S8. The $^{13}C\{^{1}H\}$ NMR spectrum of **2** ($CDCl_3$, 100 MHz). 5

Table S1. Crystal data and structure refinement for $\mathbf{L_1}$ and **1** 6

Table S2. The selected bond lengths for $\mathbf{L_1}$ 7

Table S3. The selected bond lengths for **1** 8

Figure S9. The intermolecular hydrogen bonds in crystals of $\mathbf{L_1}$ 9

Figure S10. The order of complex **1** molecules in crystal cell with the indication of intermolecular hydrogen bonding. 9

Figure S11. The CVs of compound $\mathbf{L_1}$ (CH_2Cl_2) 10

Figure S12. The CV of the electrolysis products of compound $\mathbf{L_1}$ 10

Figure S13. The CVs of compound **1** (CH_3CN) 11

Figure S14. The X-band EPR spectrum of the mixture "$\mathbf{L_1}$ + PbO_2" 11

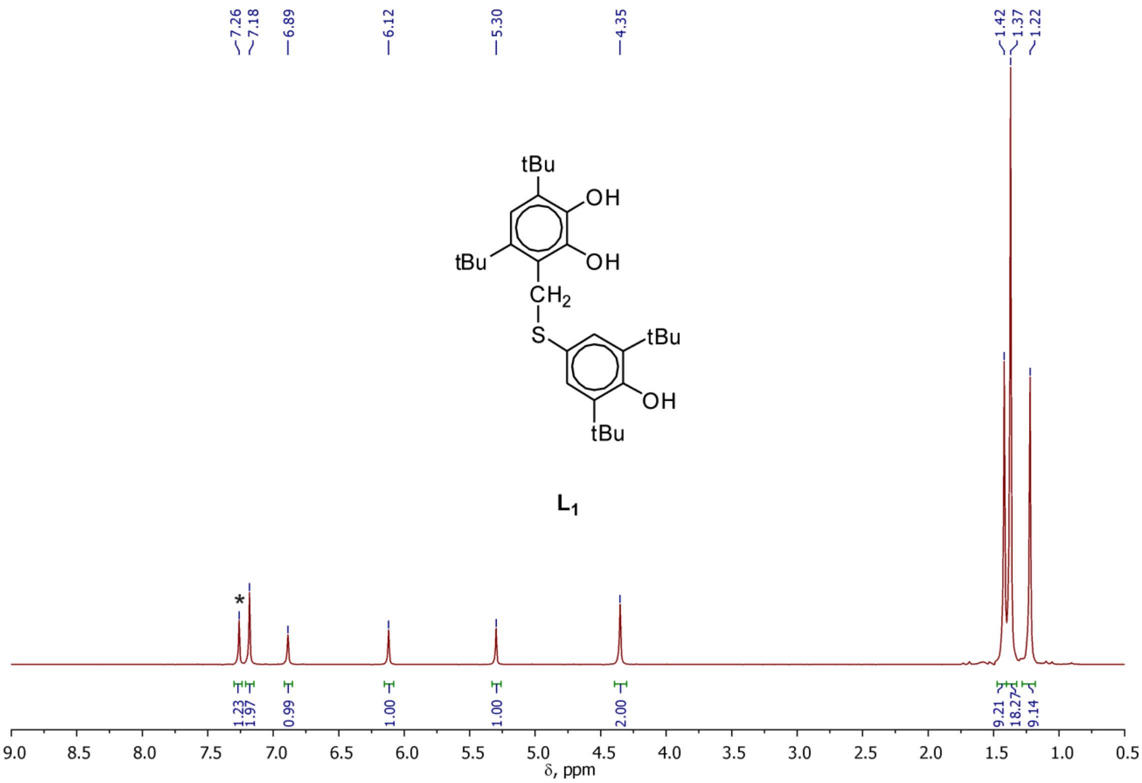

Figure S1. The ^{1}H NMR spectrum of $\mathbf{L_1}$ ($CDCl_3$, 200 MHz).

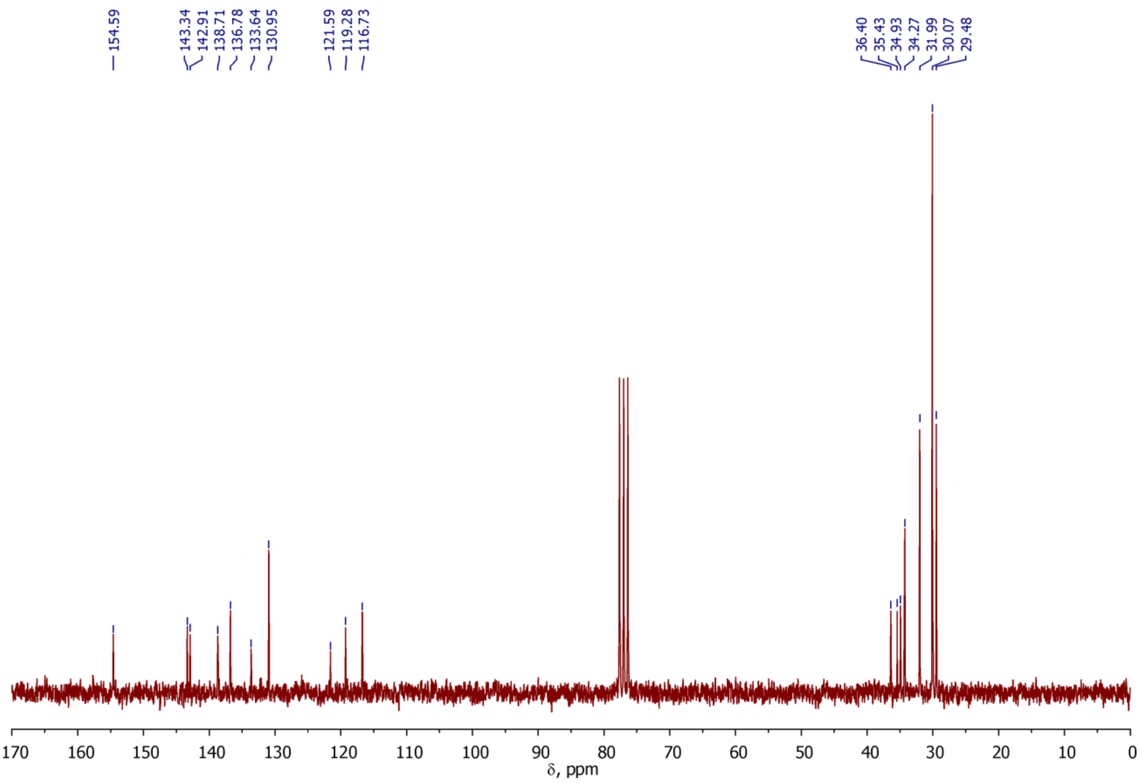

Figure S2. The $^{13}C\{^1H\}$ NMR spectrum of $\mathbf{L_1}$ ($CDCl_3$, 50 MHz).

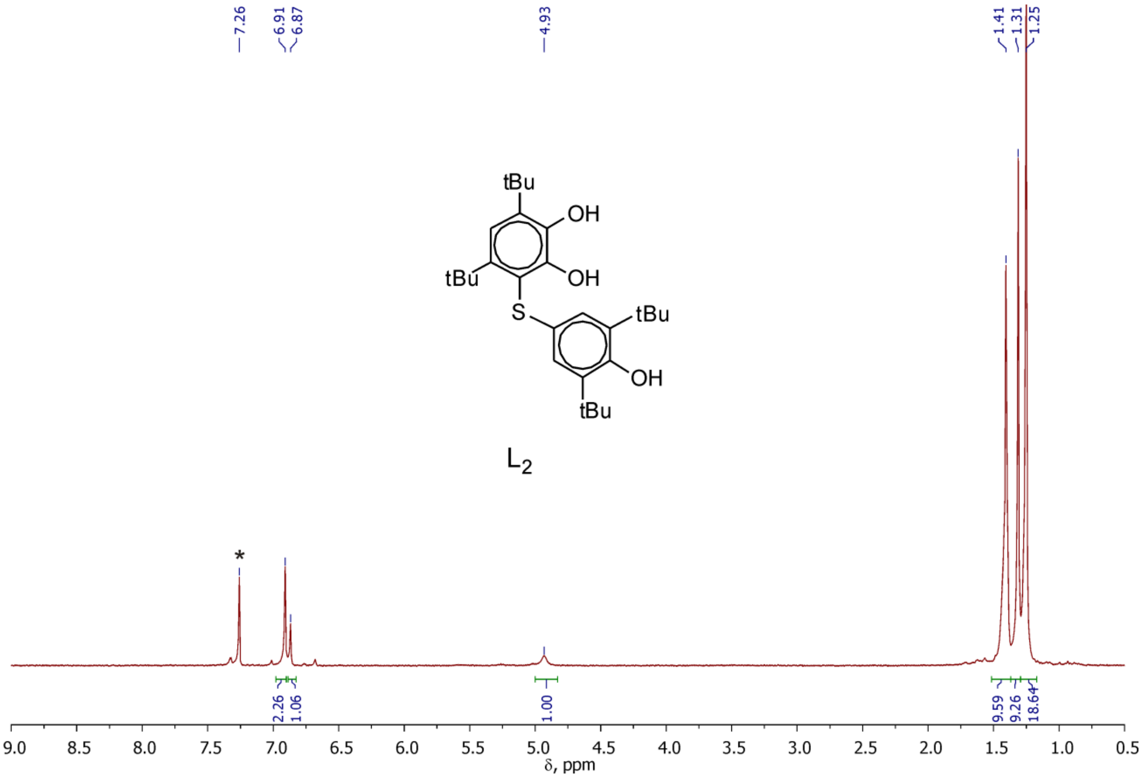

Figure S3. The ^{1}H NMR spectrum of **L_2** ($CDCl_3$, 200 MHz).

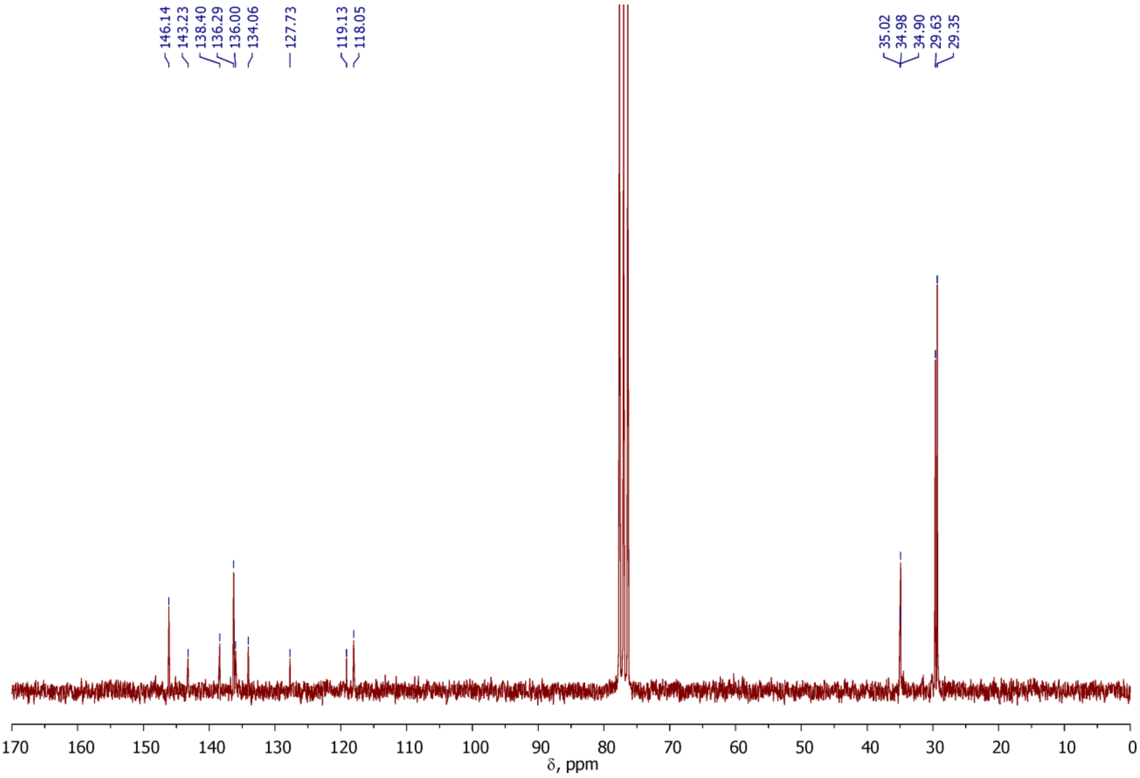

Figure S4. The ^{13}C{^{1}H} NMR spectrum of **L_2** ($CDCl_3$, 50 MHz).

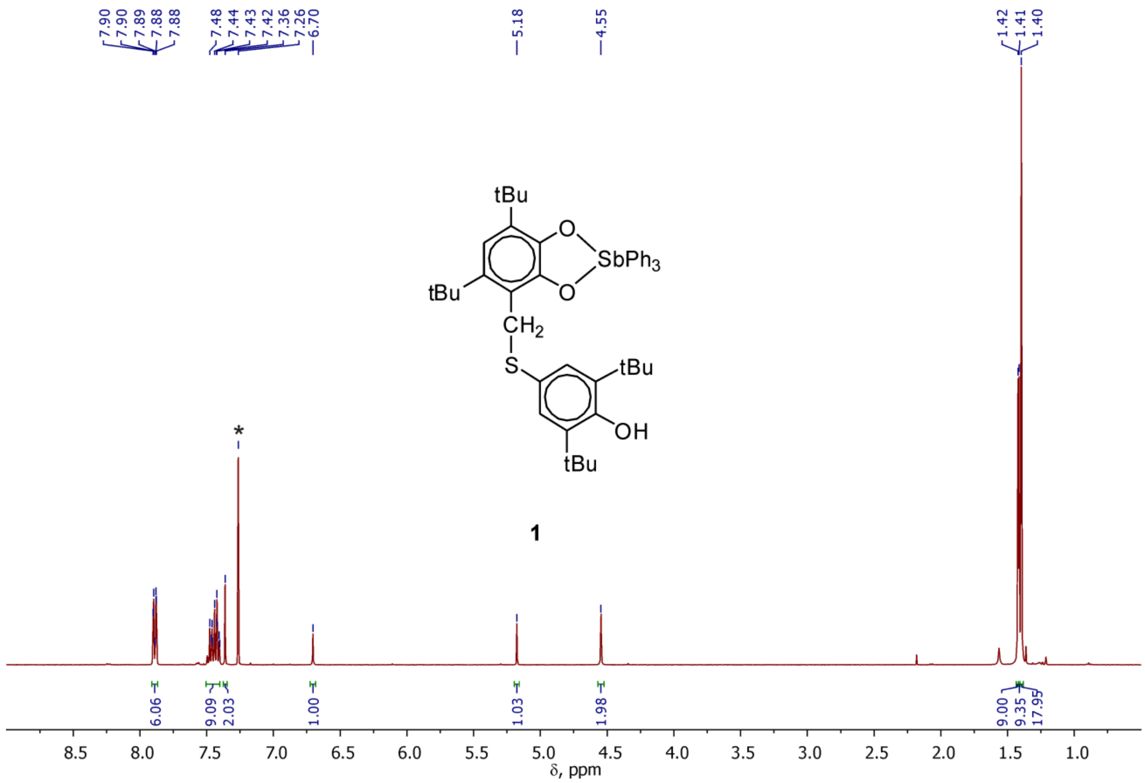

Figure S5. The ^{1}H NMR spectrum of **1** ($CDCl_3$, 400 MHz).

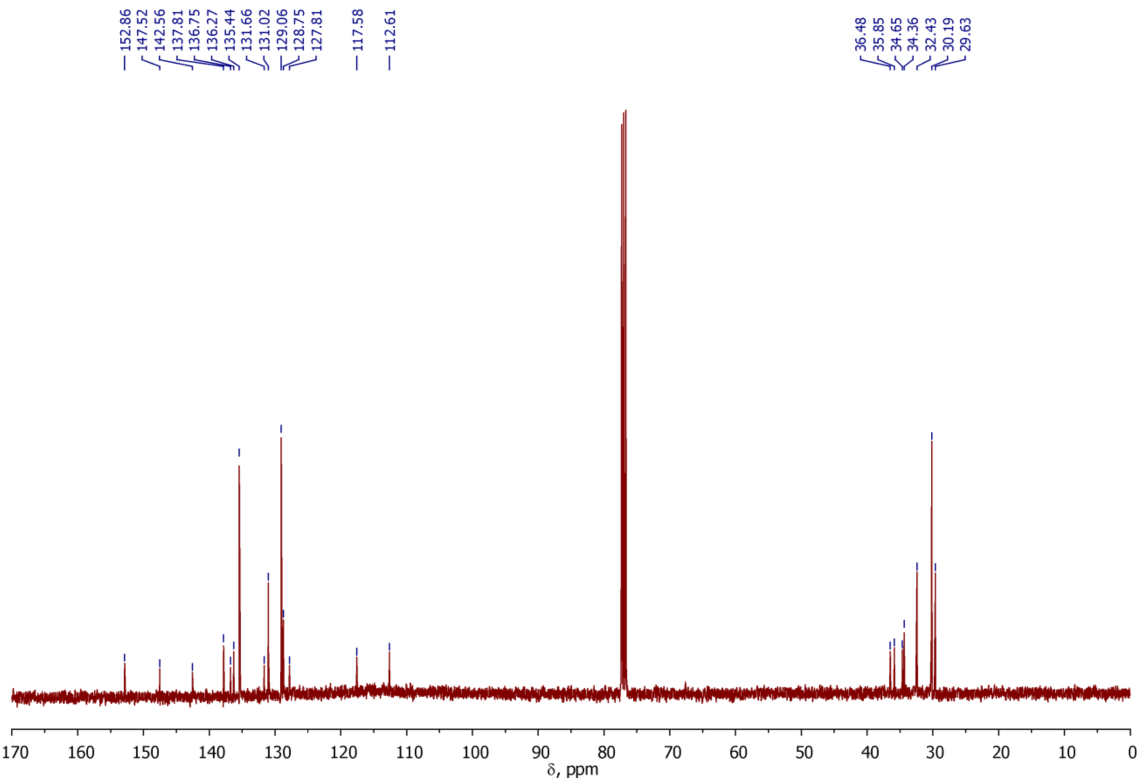

Figure S6. The ^{13}C{^{1}H} NMR spectrum of **1** ($CDCl_3$, 100 MHz).

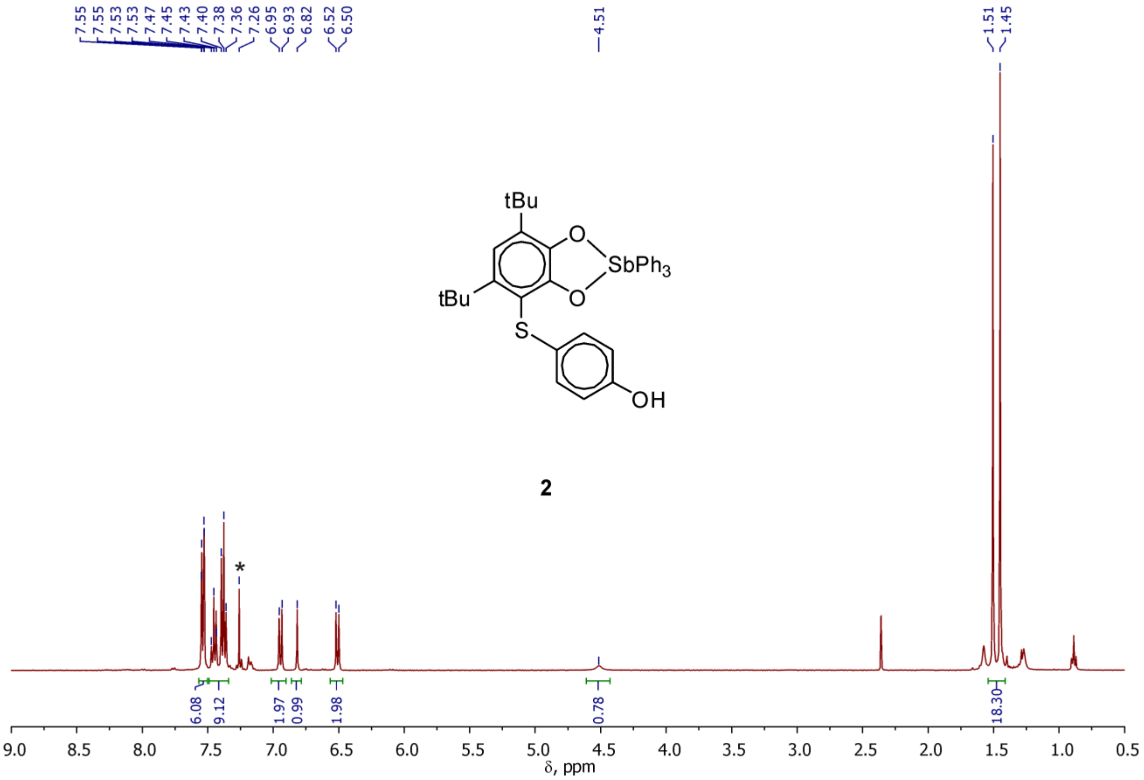

Figure S7. The ^{1}H NMR spectrum of **2** ($CDCl_3$, 400 MHz).

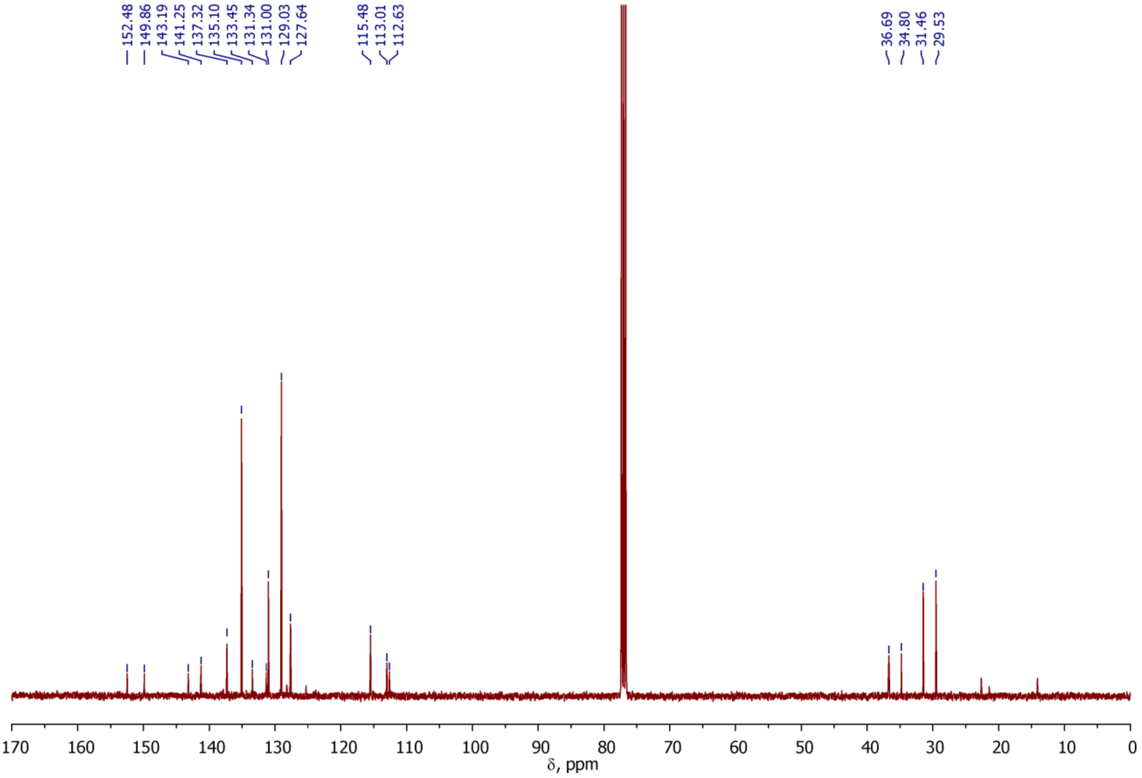

Figure S8. The $^{13}C\{^{1}H\}$ NMR spectrum of **2** ($CDCl_3$, 100 MHz).

Table S1. Crystal data and structure refinement for **L1** and **1**.

	**L_1**	**1**
Empirical formula	$C_{29}H_{44}O_3S$	$C_{47}H_{57}O_3SSb$
Formula weight	472.70	823.73
Temperature, K	100(2)	100(2)
Crystal system	Triclinic	Monoclinic
Space group	P-1	P2(1)/n
Unit cell dimensions<br>a, Å<br>b, Å<br>c, Å<br>alpha, º<br>beta, º<br>gamma, º	 10.8864(5)<br>11.5220(5)<br>12.6870(5)<br>84.5080(10)<br>69.6320(10)<br>63.2450(10)	 14.9452(8)<br>11.5228(6)<br>25.0515(14)<br>90<br>90.9770(10)<br>90
Volume, A^3	1328.61(10)	4313.5(4)
Z, Calculated density, Mg/m^3	2, 1.182	4, 1.268
Absorption coefficient mm^{-1}	0.149	0.726
F(000)	516	1720
Crystal size, mm	0.420 x 0.350 x 0.320	1.000 x 0.710 x 0.600
Θ range, deg.	1.717 - 29.127	1.945 - 25.999
Limiting indices	-14<=h<=14<br>-15<=k<=15<br>-17<=l<=17	18<=h<=18<br>-14<=k<=14<br>-30<=l<=30
Reflections collected / unique	19531 / 7116<br>[R(int) = 0.0195]	36556 / 8456<br>[R(int) = 0.0230]
Completeness to Θ = 25.242º, %	100.0 %	99.7
Absorption correction	Semi-empirical from equivalents	Semi-empirical from equivalents
Refinement method	Full-matrix least-squares on F^2	Full-matrix least-squares on F^2
Data / restraints / parameters	7116 / 0 / 322	8456 / 0 / 485
Goodness-of-fit on F^2	1.030	1.050
Final R indices [I>2sigma(I)]	R_1 = 0.0439, wR_2 = 0.1128	R_1 = 0.0281, wR_2 = 0.0678
R indices (all data)	R_1 = 0.0506, wR_2 = 0.1204	R_1 = 0.0310, wR_2 = 0.0692
Larg.diff. peak and hole, e.A^{-3}	0.472 and -0.212	0.918 and -0.298

Table S2. The selected bond lengths for $\mathbf{L_1}$.

bond	distance, Å
Sb(1)-O(1)	2.0402(13)
Sb(1)-O(2)	2.0256(13)
Sb(1)-C(30)	2.1328(19)
Sb(1)-C(36)	2.1383(19)
Sb(1)-C(42)	2.1054(19)
O(1)-C(1)	1.358(2)
O(2)-C(2)	1.366(2)
O(3)-C(19)	1.376(2)
O(3)-H(1)	0.73(3)
S(1)-C(7)	1.8365(18)
S(1)-C(16)	1.7772(19)
C(1)-C(2)	1.398(3)
C(1)-C(6)	1.393(3)
C(2)-C(3)	1.392(3)
C(3)-C(4)	1.415(3)
C(4)-C(5)	1.400(3)
C(5)-C(6)	1.401(3)
C(16)-C(17)	1.385(3)
C(16)-C(21)	1.389(3)
C(17)-C(18)	1.394(3)
C(18)-C(19)	1.408(3)
C(19)-C(20)	1.390(3).
C(20)-C(21)	

Table S3. The selected bond lengths for **1**.

bond	distance, Å
Sb(1)-O(1)	2.0402(13)
Sb(1)-O(2)	2.0256(13)
Sb(1)-C(30)	2.1328(19)
Sb(1)-C(36)	2.1383(19)
Sb(1)-C(42)	2.1054(19)
O(1)-C(1)	1.358(2)
O(2)-C(2)	1.366(2)
O(3)-C(19)	1.376(2)
O(3)-H(1)	0.73(3)
S(1)-C(7)	1.8365(18)
S(1)-C(16)	1.7772(19)
C(1)-C(2)	1.398(3)
C(1)-C(6)	1.393(3)
C(2)-C(3)	1.392(3)
C(3)-C(4)	1.415(3)
C(4)-C(5)	1.400(3)
C(5)-C(6)	1.401(3)
C(16)-C(17)	1.385(3)
C(16)-C(21)	1.389(3)
C(17)-C(18)	1.394(3)
C(18)-C(19)	1.413(3)
C(19)-C(20)	1.408(3)
C(20)-C(21)	1.390(3)

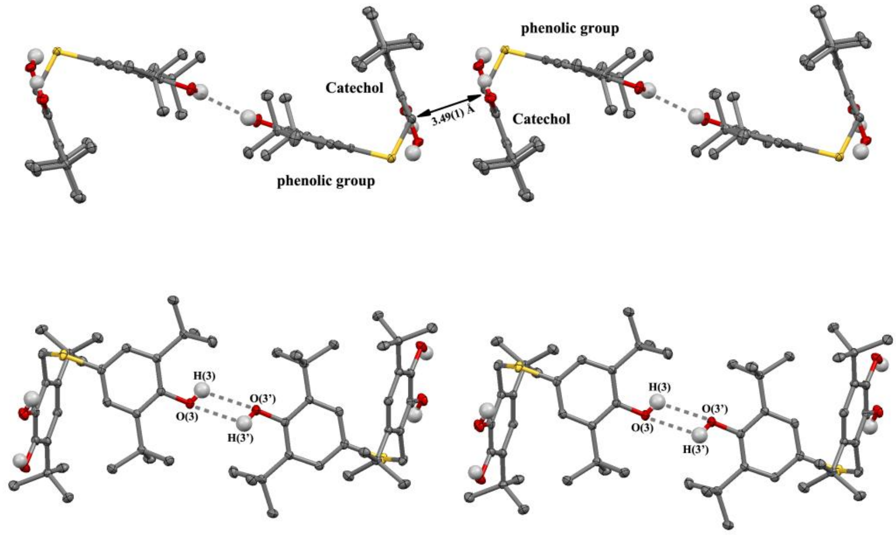

Figure S9. The intermolecular hydrogen bonds in crystals of $\mathbf{L_1}$.

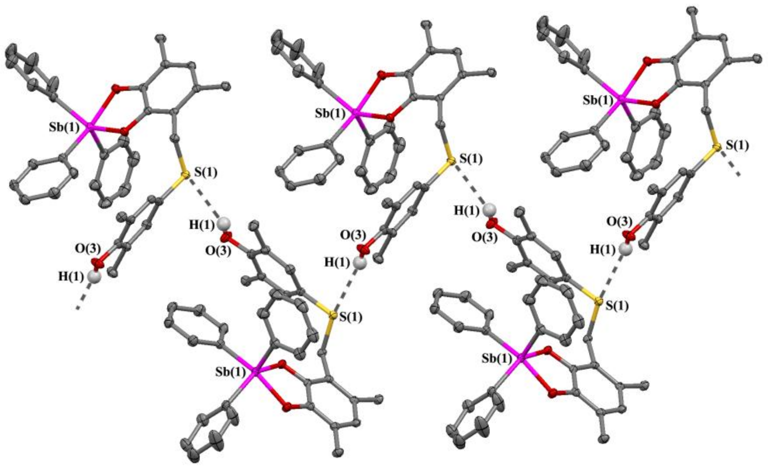

Figure S10. The order of complex **1** molecules in crystal cell with the indication of intermolecular hydrogen bonding. The methyl groups of tert-butyls and hydrogen atoms excepting atoms H(1) are omitted for clarity. The ellipsoids of 50% probability.

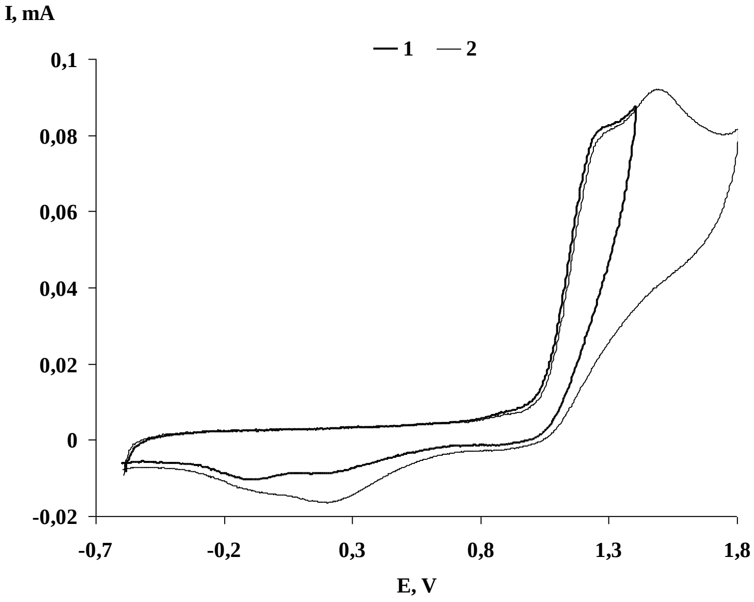

Figure S11. The CVs of compound $\mathbf{L}_1$ (in the potential switch from -0.6 to 1.4 V – curve 1; in the potential switch from -0.6 to 1.8 V – curve 2) (CH_2Cl_2, C = 3 mM, 0.15 M TBAP, scan rate 200 mV·s^{-1}).

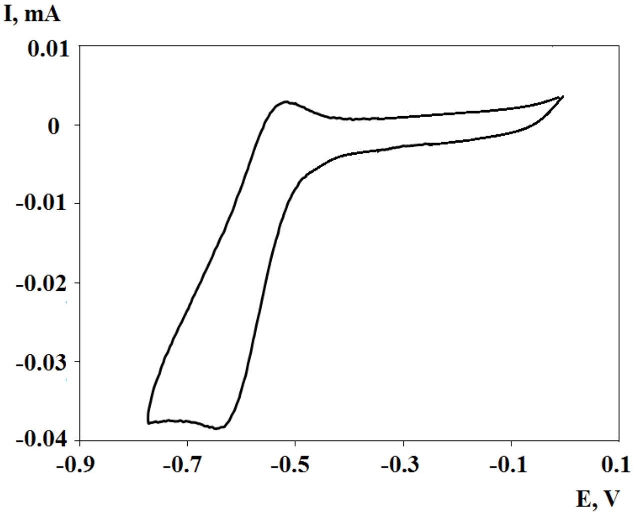

Figure S12. The CV of the electrolysis products of compound $\mathbf{L_1}$ in the potential switch from 0.0 to - 0.78 V (MeCN, 90 min, E = 1.2 V, C = 2 mM, 0.15 M TBAP, scan rate 200 mV·s^{-1}).

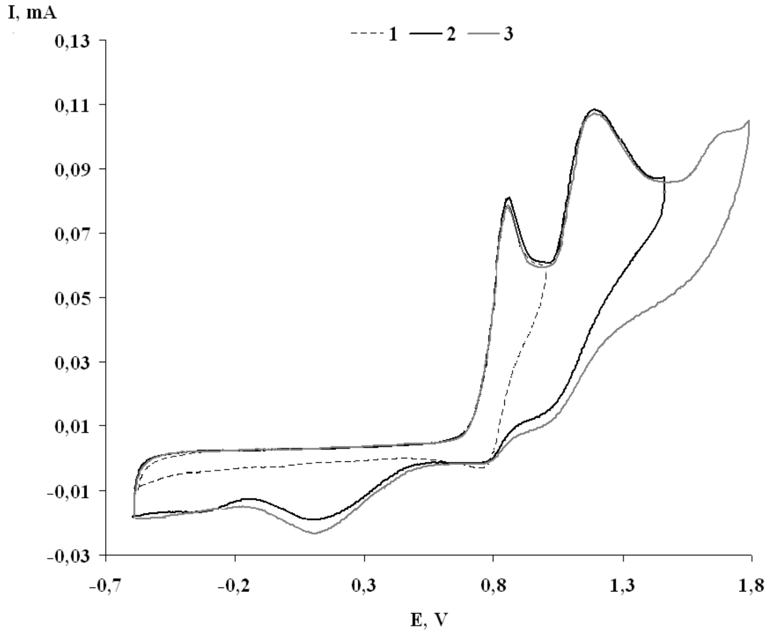

Figure S13. The CVs of compound **1** (in the potential switch from -0.5 to 1.0 V – curve 1; in the potential switch from -0.5 to 1.5 V – curve 2; in the potential switch from -0.5 to 1.8 V – curve 3) (CH_3CN, C = 3 mM, 0.15 M TBAP, scan rate 200 $mV{\cdot}s^{-1}$).

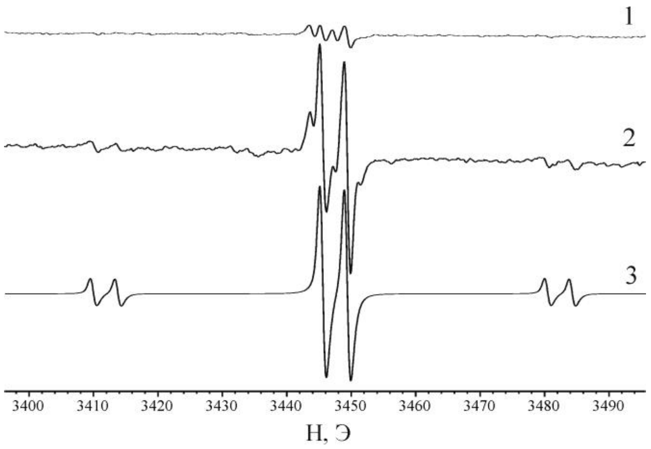

Figure S14. The EPR spectrum of the mixture "$\mathbf{L_1}$ + PbO_2" in toluene immediately after mixing the reagents (spectrum 1), after heating at 60°C for 15 minutes (spectrum 2), and simulated EPR spectrum (WinEPR SimFonia 1.25) with parameters g_i = 2.0009, $a_i(^1H)$ = 3.85 G, $a_i(^{207}Pb)$ = 70.5 G (spectrum 3).
